# Supplementary material for: Gasdermin E dictates inflammatory responses by controlling the mode of neutrophil death
Source: Nat Commun. 2024 Jan 9;15:386. doi: 10.1038/s41467-023-44669-y (PMC10776763; doi:10.1038/s41467-023-44669-y)
Supplement: Supplementary file 1 — Supplementary Information [file 41467_2023_44669_MOESM1_ESM.pdf]

Supplemental information for

Gasdermin E dictates inflammatory responses by controlling the mode of neutrophil death

Fengxia Ma[1#*], Laxman Ghimire[2#], Qian Ren[1#], Yuping Fan[1], Tong Chen[1], Arumugam Balasubramanian[2], Alan Hsu[2], Fei Liu[2], Hongbo Yu[3], Xuemei Xie[2], Rong Xu[2], Hongbo R Luo[2*]

The PDF file includes:

Supplemental Figures

Fig.S1. Expression of gasdermin family proteins in neutrophils.
Fig.S2. Generation of GSDME whole-body KO mice.
Fig.S3. Analysis of neutrophil death after fixation and staining with SYTOX Orange.
Fig.S4. Quantification of the absolute number of neutrophils by flow cytometry using counting beads.
Fig.S5. GSDME disruption abolished lytic cell death and skewed the death program toward apoptosis in neutrophils isolated from inflamed peritoneal cavities.
Fig.S6. GSDME disruption skewed neutrophil death to apoptosis in the presence of proinflammatory cytokines.
Fig.S7. GSDMD deficiency delayed neutrophil death programs but did not alter the mode of neutrophil death.
Fig.S8. Cleavage of GSDME during the death of human neutrophils.
Fig.S9. GSDME disruption in neutrophils did not affect overall neutrophil survival *in vivo.*
Fig.S10. Impact of whole-body GSDME knockout on immune cell numbers and trafficking to inflammatory sites.
Fig.S11. Generation of neutrophil-specific *Gsdme* conditional KO mice (*Mrp8-cre(+)/Gsdme*$^{\Delta/\Delta}$).
Fig.S12. Impact of neutrophil-specific GSDME knockout on immune cell numbers and trafficking to inflammatory sites.
Fig.S13. GSDME disruption attenuated inflammation and alleviated lung injury during LPS-induced pneumonia.
Fig.S14. GSDME disruption attenuated inflammation and alleviated lung injury in acid aspiration pneumonitis.
Fig.S15. GSDMD and GSDME exert different functions in neutrophil spontaneous death.

Supplemental Methods

Supplemental Movies

Movie S1
Movie S2

Supplemental References

Other Supplementary Material for this manuscript includes the following:

Source Data File (Microsoft Excel format). Raw data for all figures.

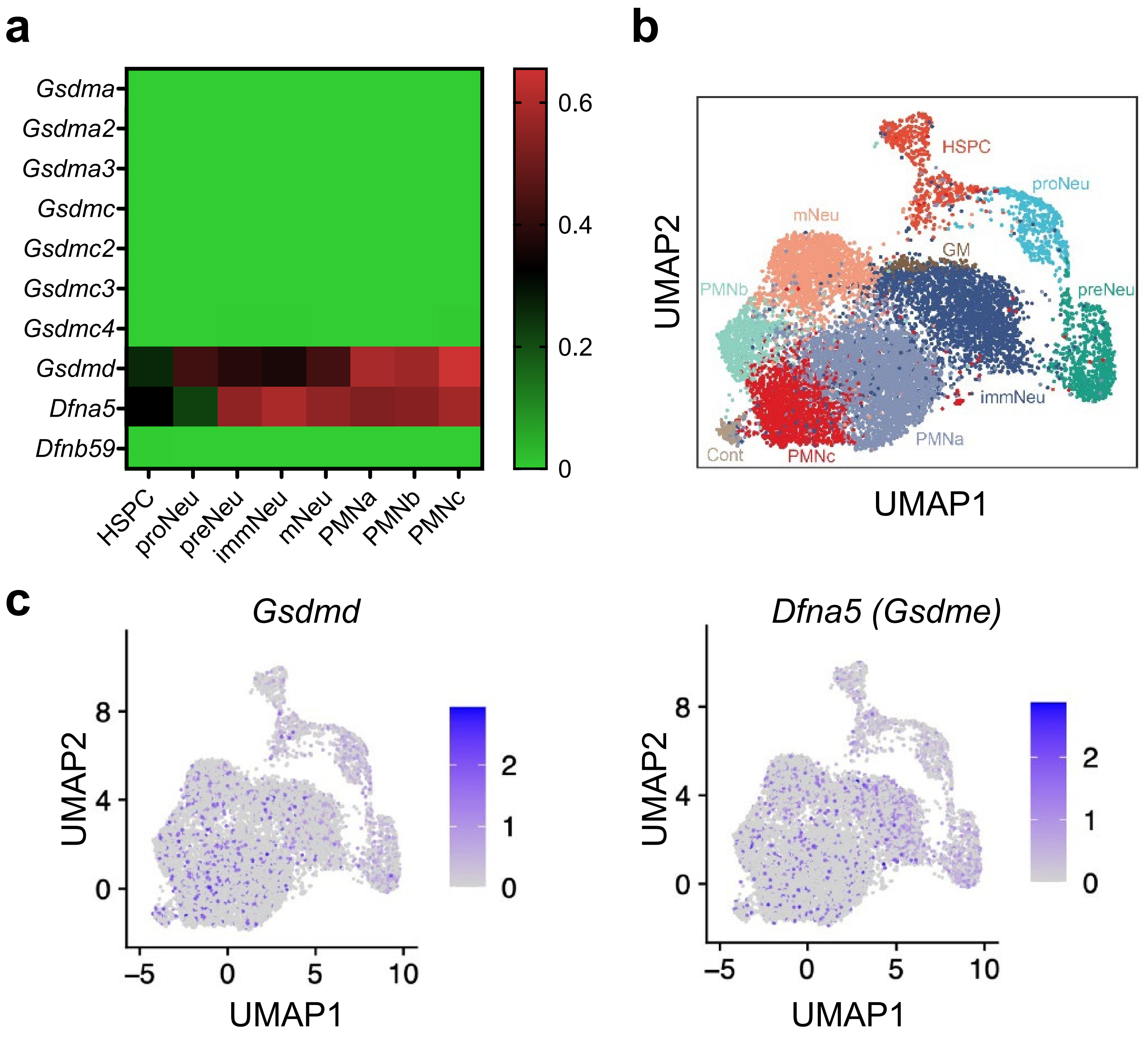

a
Gsdma
Gsdma2
Gsdma3
Gsdmc
Gsdmc2
Gsdmc3
Gsdmc4
Gsdmd
Dfna5
Dfnb59
HSPC
proNeu
preNeu
immNeu
mNeu
PMNa
PMNb
PMNc
0.6
0.4
0.2
0
b
UMAP2
UMAP1
HSPC
proNeu
mNeu
GM
PMNb
preNeu
immNeu
PMNa
Cont
PMNc
c
Gsdmd
Dfna5 (Gsdme)
UMAP2
UMAP1
8
4
0
−5
0
5
10
2
1
0

Fig.S1. Expression of gasdermin family proteins in neutrophils.

Expression of gasdermin family proteins was analyzed based on single-cell mRNA sequencing (scRNA-Seq) of mouse bone marrow (BM), peripheral blood (PB), and spleen (SP) hematopoietic stem and progenitor cells (HSPC) and neutrophils. scRNA-Seq analysis was performed as previously described [1]. Briefly, the quality of sequencing reads was evaluated using FastQC and MultiQC. Cell Ranger v2.2.0 was used to align the sequencing reads (fastq) to the mm10 mouse transcriptome and quantify the expression of transcripts in each cell. This pipeline resulted in a gene expression matrix for each sample, which records the number of UMIs for each gene associated with each cell barcode. All downstream analyses were implemented using R v3.5.2 and the package Seurat v2.3.4 117. Dimension reduction was performed at three stages of the analysis: the selection of variable genes, principal component analysis (PCA), and uniform manifold approximation and projection (UMAP). For unsupervised clustering, we performed the FindClusters function to cluster cells using the Louvain algorithm based on the same PCs as RunUMAP function.

(a) mRNA expression of gasdermin family members in HSPCs and neutrophils at different developmental stages. HSPC: hematopoietic stem and progenitor, proNeu: neutrophil progenitors, preNeu: neutrophil precursor, immNeu: immature neutrophil, mNeu: mature neutrophil, PMN: polymorphonuclear neutrophil.
(b) Uniform manifold approximation and projection (UMAP) of HSPCs and neutrophils at different differentiation stages[1].
(c) Expression patterns of *Gsdmd* and *Gsdme* projected on the UMAP plot.
Sequencing data related to **Fig.S1** have been deposited at NCBI GEO depository and are accessible with the accession number GSE137540.

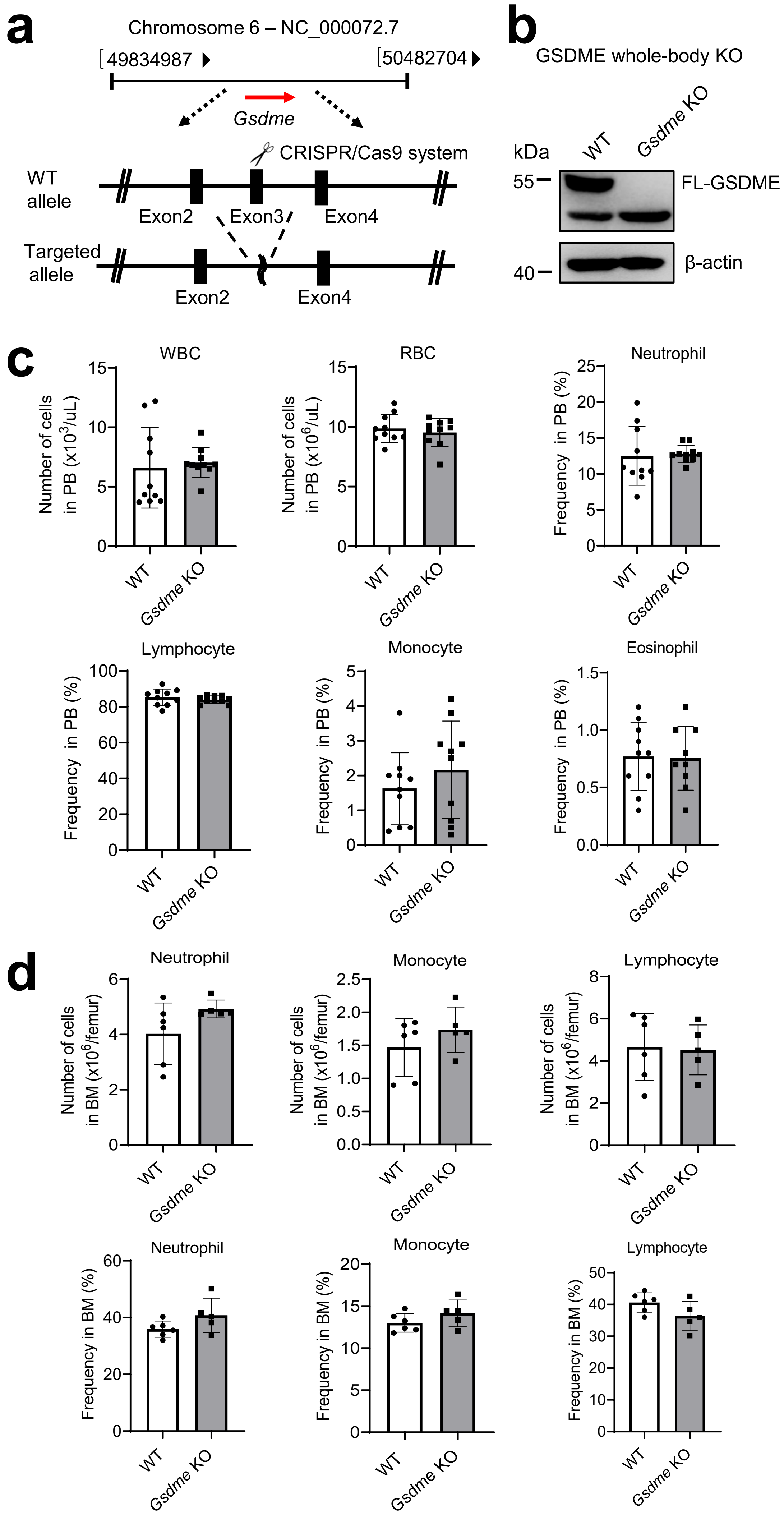

a
Chromosome 6 – NC_000072.7
49834987
50482704
Gsdme
CRISPR/Cas9 system
WT allele
Exon2
Exon3
Exon4
Targeted allele
Exon2
Exon4
b
GSDME whole-body KO
kDa
WT
Gsdme KO
55
40
FL-GSDME
β-actin
c
WBC
RBC
Neutrophil
Lymphocyte
Monocyte
Eosinophil
Number of cells in PB (x10^3/uL)
Number of cells in PB (x10^6/uL)
Frequency in PB (%)
WT
Gsdme KO
d
Neutrophil
Monocyte
Lymphocyte
Number of cells in BM (x10^6/femur)
Frequency in BM (%)
WT
Gsdme KO

Fig.S2. Generation of GSDME whole-body KO mice.

(a) Schematic showing the strategy to generate *Gsdme* KO mice using the CRISPR/Cas9 system.

(b) Confirmation of GSDME deletion in neutrophils from *Gsdm*e KO mice by western blotting.

(c) Total and differential leukocyte counts along with total erythrocyte count in the peripheral blood of WT and *Gsdme* KO mice. All data are represented as mean ± SD, n = 10 mice.

(d) Absolute number and percentage of neutrophils, monocytes, and lymphocytes in the bone marrow of WT and *Gsdme* KO neutrophils. All data are represented as mean ± SD, n = 6 mice.

Source data are provided as a Source Data file.

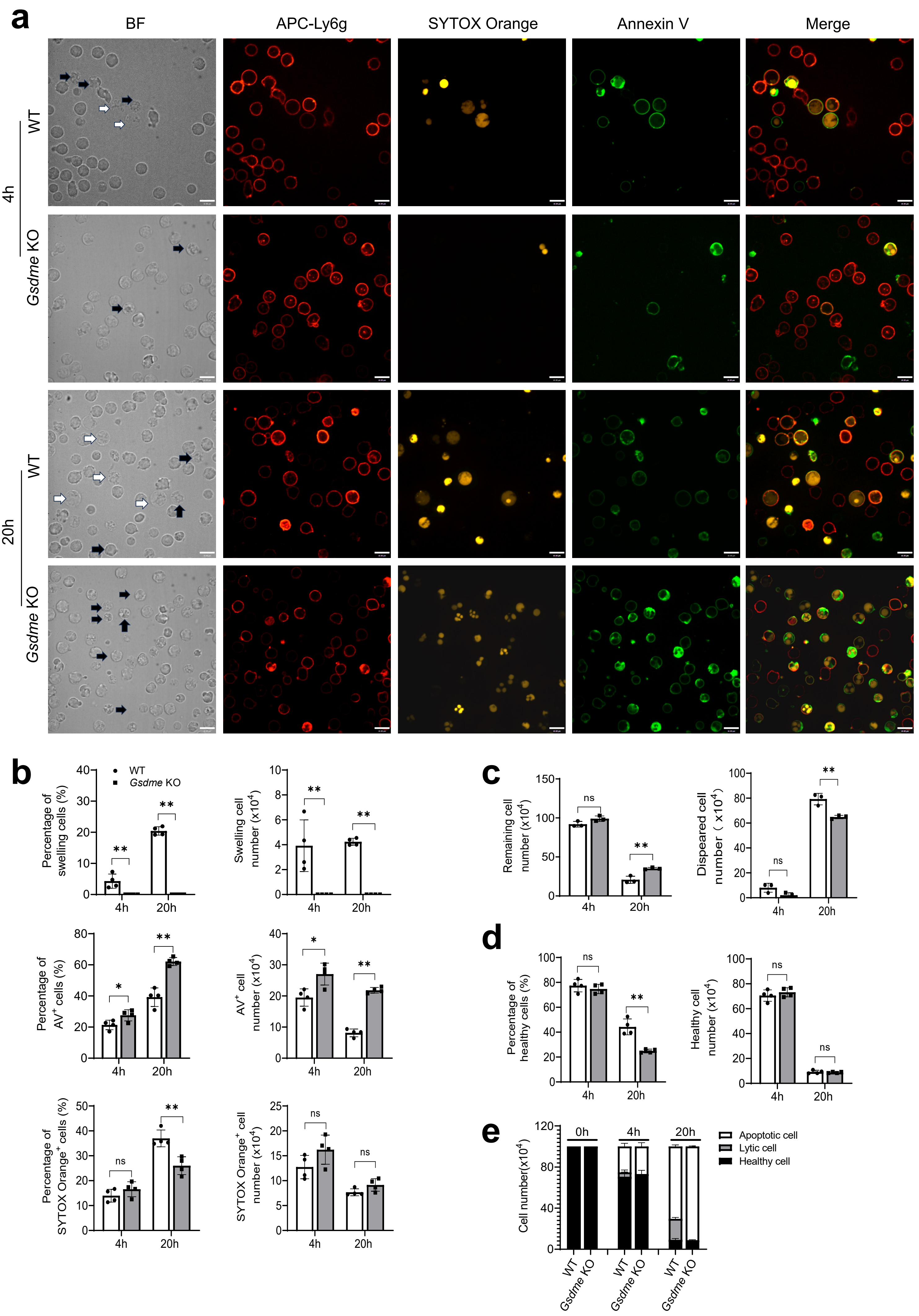

a
BF
APC-Ly6g
SYTOX Orange
Annexin V
Merge
4h
WT
Gsdme KO
20h
WT
Gsdme KO
b
WT
Gsdme KO
Percentage of swelling cells (%)
Swelling cell number (x10^4)
Percentage of AV+ cells (%)
AV+ cell number (x10^4)
Percentage of SYTOX Orange+ cells (%)
SYTOX Orange+ cell number (x10^4)
4h
20h
c
Remaining cell number (x10^4)
Dispeared cell number（x10^4）
d
Percentage of healthy cells (%)
Healthy cell number (x10^4)
e
Cell number(x10^4)
0h
4h
20h
Apoptotic cell
Lytic cell
Healthy cell

Fig.S3. Analysis of neutrophil death after fixation and staining with SYTOX Orange. At each indicated time point, neutrophils were stained with APC-Ly6g, SYTOX Orange dye and FITC-Annexin V at 4°C for 20 minutes, followed by fixation with 4% paraformaldehyde at 4°C for another 15 minutes. Images were captured using a confocal microscope with a 60x objective.

(a) Morphological analysis of neutrophils undergoing apoptosis and lytic cell death at the indicated time points. The white arrow heads indicate swollen or “puffed” cells (lytic cell death). The black arrow heads indicate apoptotic cells. BF, bright field. Results are representative of at least three independent experiments.

(b) The percentage and number of swollen, AV-positive, and SYTOX Orange -positive cells at the indicated time points. All data are represented as mean ± SD. n=4 independent repeats, *P < 0.05, **P < 0.01, ns, non-significant.

(c) The number of intact neutrophils remaining at each indicated time point was counted using a hemocytometer. The number of disappearing neutrophils was calculated as the initial cell count (time 0) minus the remaining cell count at each time point. All data are represented as mean ± SD. n=3 independent repeats, *P < 0.05, **P < 0.01. ns, non-significant.

(d) The percentage and number of healthy neutrophils at the indicated time points.SO, SYTOX Orange. All data are represented as mean ± SD. n=4 independent repeats, *P < 0.05, **P < 0.01, ns, non-significant.

(e) The number of healthy neutrophils and neutrophils undergoing apoptosis or lytic cell death at the indicated time points. The mode of neutrophil death was determined based on the morphological analysis (a). To calculate the percentages, at least 200 cells were tracked. All data are represented as mean± SD (n=4 independent repeats).

Source data are provided as a Source Data file.

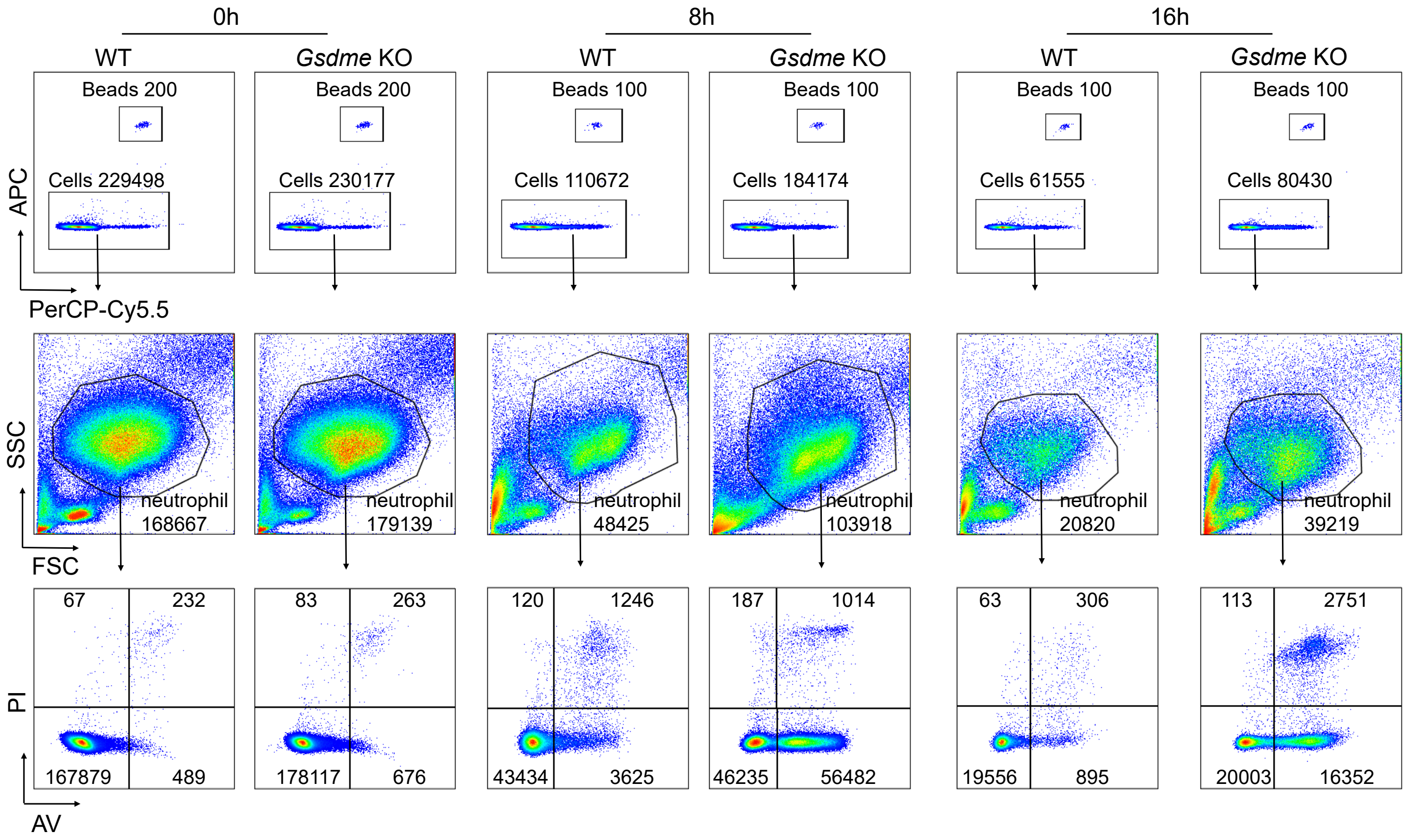
0h
8h
16h
WT
Gsdme KO
WT
Gsdme KO
WT
Gsdme KO
Beads 200
Beads 200
Beads 100
Beads 100
Beads 100
Beads 100
Cells 229498
Cells 230177
Cells 110672
Cells 184174
Cells 61555
Cells 80430
APC
PerCP-Cy5.5
SSC
FSC
neutrophil 168667
neutrophil 179139
neutrophil 48425
neutrophil 103918
neutrophil 20820
neutrophil 39219
PI
AV
67
232
167879
489
83
263
178117
676
120
1246
43434
3625
187
1014
46235
56482
63
306
19556
895
113
2751
20003
16352

Fig.S4. Quantification of the absolute number of neutrophils by flow cytometry using counting beads. Bone marrow-derived neutrophils from WT and *Gsdme* KO mice were cultured as described in **Fig.2**. At the indicated time points, cells were harvested and stained with Annexin V and PI to enumerate healthy and dying neutrophil numbers using counting beads. The upper panel shows the gating of cells and beads, the middle panel depicts the gating of neutrophils, and the lower panel shows the absolute numbers of neutrophils in each quadrant. AV, Annexin V; PI, propidium iodide. The flow cytometric pictures are representative of at least three independent experiments.

Source data are provided as a Source Data file.

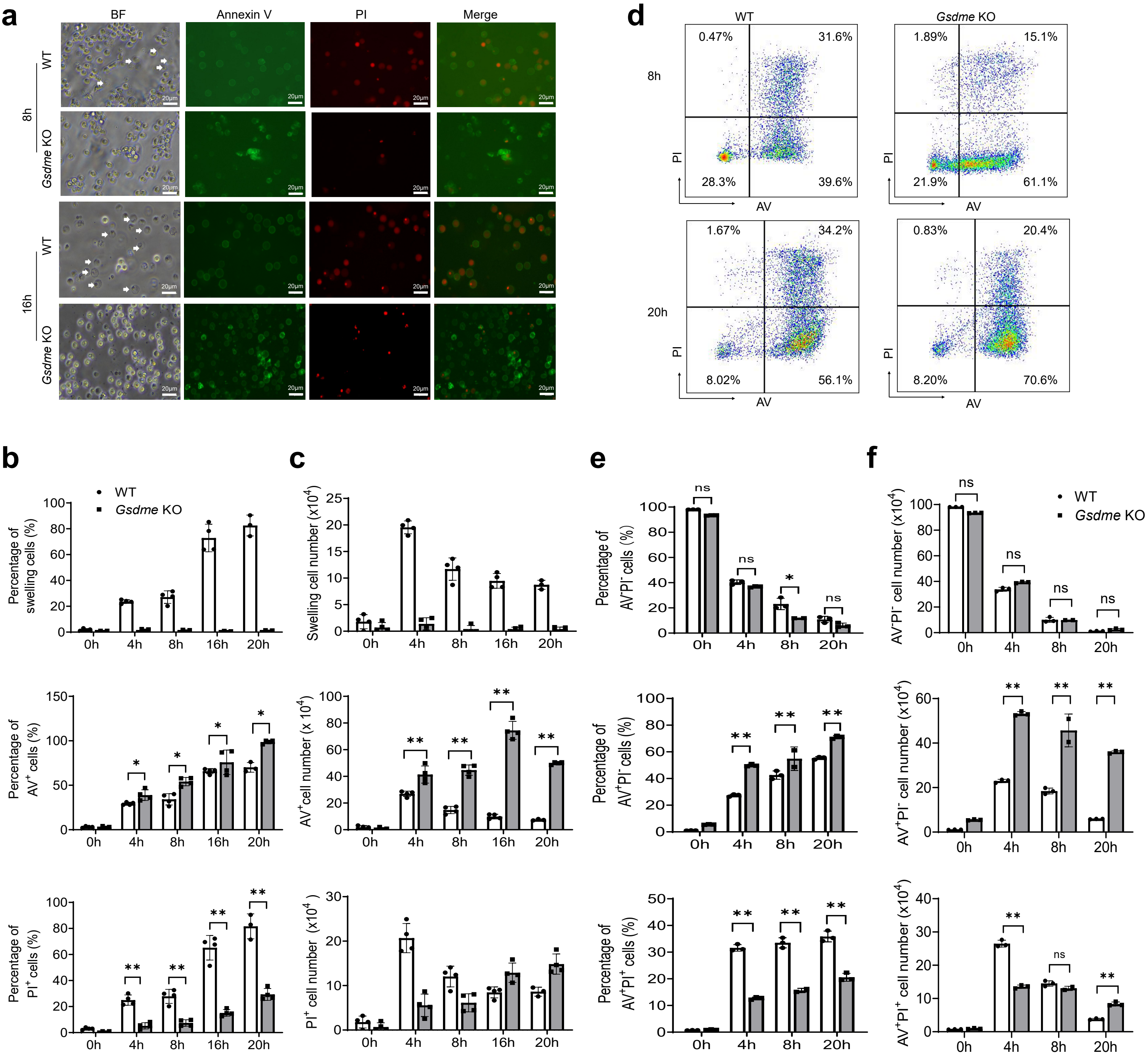
a
BF
Annexin V
PI
Merge
8h
WT
Gsdme KO
16h
20μm
d
WT
Gsdme KO
0.47%
31.6%
28.3%
39.6%
1.89%
15.1%
21.9%
61.1%
1.67%
34.2%
8.02%
56.1%
0.83%
20.4%
8.20%
70.6%
20h
PI
AV
b
c
e
f
WT
Gsdme KO
Percentage of swelling cells (%)
Swelling cell number (x10⁴)
Percentage of AV⁺PI⁻ cells (%)
AV⁻PI⁻ cell number (x10⁴)
Percentage of AV⁺ cells (%)
AV⁺cell number (x 10⁴)
Percentage of AV⁺PI⁻ cells (%)
AV⁺PI⁻ cell number (x10⁴)
Percentage of PI⁺ cells (%)
PI⁺ cell number (x10⁴)
Percentage of AV⁺PI⁺ cells (%)
AV⁺PI⁺ cell number (x10⁴)
0h
4h
8h
16h
20h
ns
*
**

Fig.S5. GSDME disruption abolished lytic cell death and skewed the death program toward apoptosis in neutrophils isolated from inflamed peritoneal cavities. Peritoneal neutrophils were isolated from thioglycolate (TG)-challenged WT and *Gsdme* KO mice. Neutrophil spontaneous death was investigated by imaging and flow cytometry as described in Fig.1 and Fig.2, respectively.

(a) Representative images of aging neutrophils at indicated time points. The white arrow heads indicate swollen or “puffed” cells (lytic cell death). PI, propidium iodide; BF, bright field. Results are representative of at least three independent experiments.

(b,c) The percentage and number of swollen, Annexin V (AV)-positive, and PI-positive cells at the indicated time points. All data are represented as mean ± SD. n = 4 independent repeats, $*P < 0.05$, $**P < 0.01$.

(d) Representative flow cytometric pictures of peritoneal neutrophils undergoing spontaneous cell death. Purified neutrophils from WT and *Gsdme* KO mice were cultured for the indicated times and stained with AV and PI. Results are representative of at least 3 independent experiments.

(e,f) The percentage and number of AV^-PI^-, AV^+PI^-, and AV^+PI^+ cells at the indicated time points. All data are represented as mean ± SD. n=3 independent repeats, $**P < 0.01$, ns, non-significant ($P > 0.05$). GSDME disruption did not alter the number of heathy (AV^-PI^-) neutrophils remaining at each time point.

Source data are provided as a Source Data file.

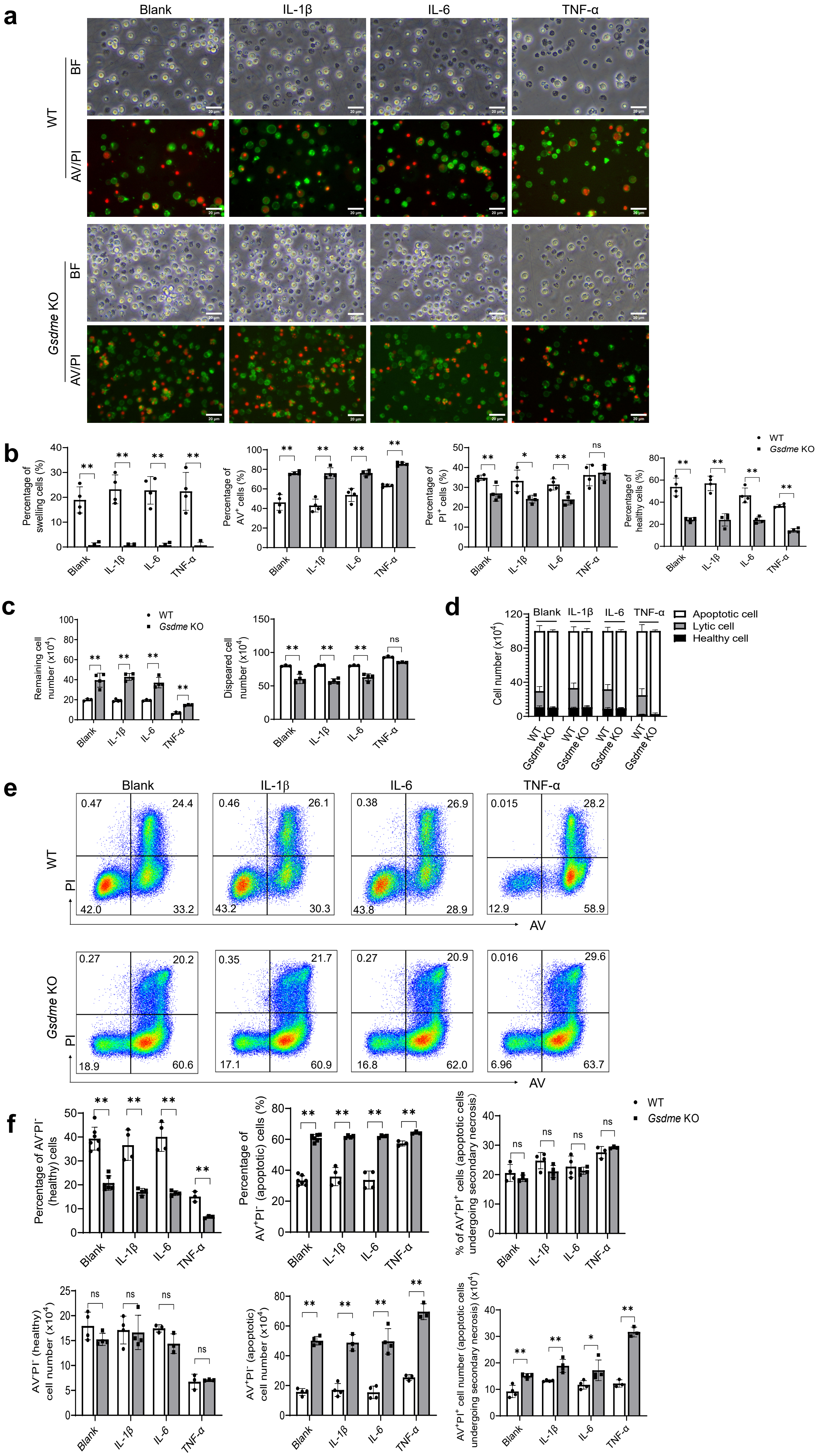

a
Blank
IL-1β
IL-6
TNF-α
WT
BF
AV/PI
Gsdme KO
BF
AV/PI
20 μm
b
Percentage of swelling cells (%)
Percentage of AV+ cells (%)
Percentage of PI+ cells (%)
Percentage of healthy cells (%)
WT
Gsdme KO
Blank
IL-1β
IL-6
TNF-α
**
*
ns
c
Remaining cell number (x10^4)
Dispeared cell number (x10^4)
WT
Gsdme KO
d
Cell number (x10^4)
Apoptotic cell
Lytic cell
Healthy cell
WT
Gsdme KO
e
Blank
IL-1β
IL-6
TNF-α
WT
PI
AV
0.47
24.4
42.0
33.2
0.46
26.1
43.2
30.3
0.38
26.9
43.8
28.9
0.015
28.2
12.9
58.9
Gsdme KO
0.27
20.2
18.9
60.6
0.35
21.7
17.1
60.9
0.27
20.9
16.8
62.0
0.016
29.6
6.96
63.7
f
Percentage of AV-PI- (healthy) cells
Percentage of AV+PI- (apoptotic) cells (%)
% of AV+PI+ cells (apoptotic cells undergoing secondary necrosis)
WT
Gsdme KO
AV-PI- (healthy) cell number (x10^4)
AV+PI- (apoptotic) cell number (x10^4)
AV+PI+ cell number (apoptotic cells undergoing secondary necrosis) (x10^4)

Fig.S6. GSDME disruption skewed neutrophil death to apoptosis in the presence of proinflammatory cytokines.

(a) Representative images of neutrophils undergoing apoptosis and lytic cell death. Neutrophils were treated with 10 ng/ml IL-1β, IL-6 or TNF-α for 20h. Results are representative of at least three independent experiments.

(b) The percentage swollen, AV^+, PI^+, and healthy (AV^-PI^-) cells. All data are represented as mean ± SD. n=4 independent repeats, *P < 0.05, **P < 0.01, ns, non-significant. The analysis was performed as described in **Fig.1.**

(c) The number of intact neutrophils remaining was counted using a hemocytometer. The number of disappearing neutrophils was calculated as the initial cell count (time 0) minus the remaining cell count at each time point. All data are represented as mean ± SD. n=4 independent repeats, *P < 0.05, **P < 0.01. ns, non-significant.

(d) The absolute number of healthy neutrophils and neutrophils undergoing apoptosis or lytic cell death. The analysis was performed as described in **Fig.1g**. All data are represented as mean ± SD (n=4 independent repeats)

(e) Representative flow cytometry plots of neutrophils undergoing cell death. Neutrophils were treated with 10 ng/ml IL-1β, IL-6 or TNF-α for 20h. Results are representative of at least three biological replicates.

(f) The percentage and number of AV^-PI^-, AV^+PI^-, and AV^+PI^+ cells at the indicated stimulation. The analysis was performed as described in **Fig.2b-c**. All data are represented as mean ± SD. n=3-4 independent repeats, *P < 0.05, **P < 0.01, ns, non-significant.

Source data are provided as a Source Data file.

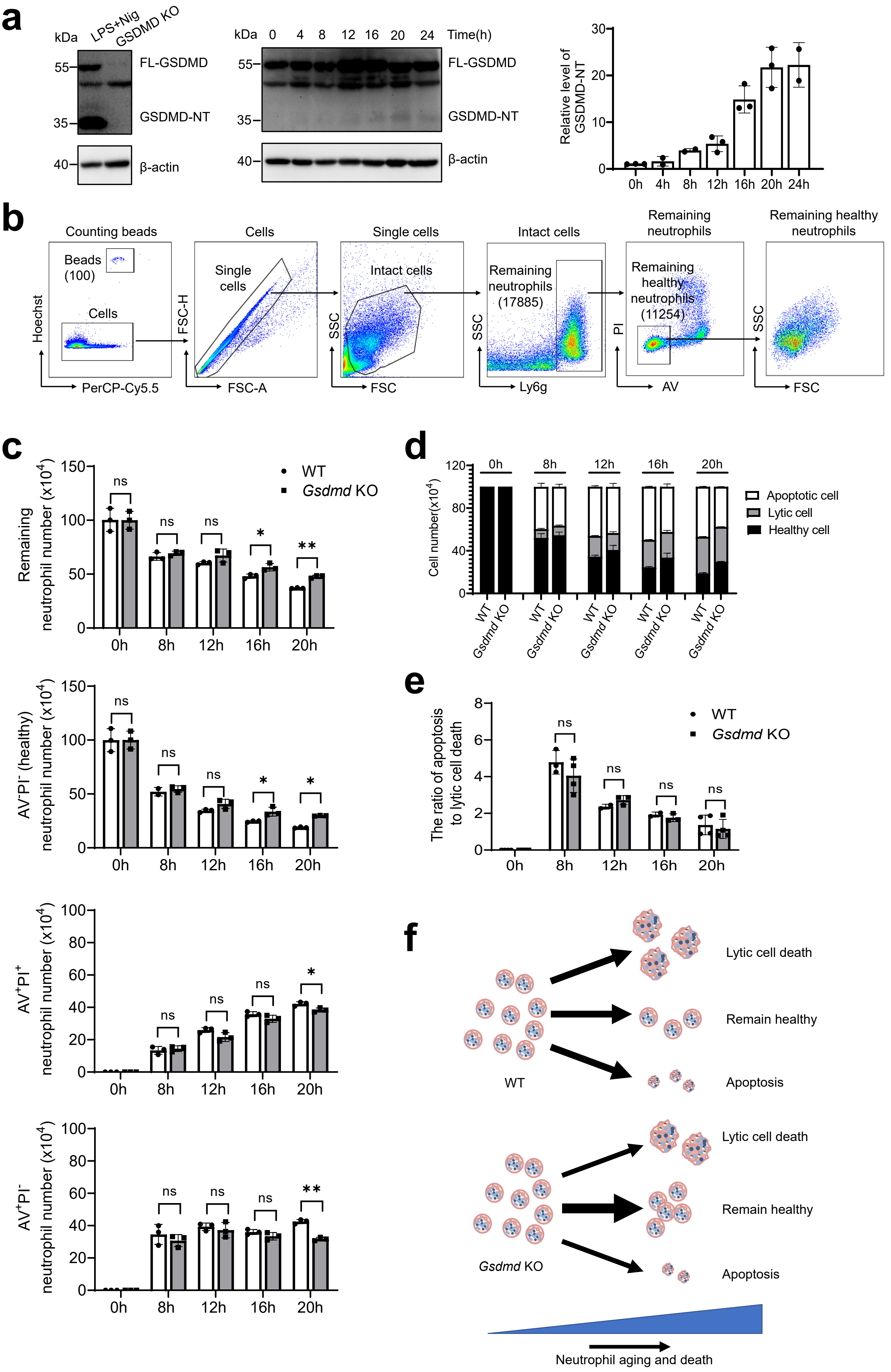
a
kDa
LPS+Nig
GSDMD KO
55
35
40
FL-GSDMD
GSDMD-NT
β-actin
kDa 0 4 8 12 16 20 24 Time(h)
Relative level of GSDMD-NT
0h 4h 8h 12h 16h 20h 24h
b
Counting beads
Beads (100)
Cells
Hoechst
PerCP-Cy5.5
Single cells
FSC-H
FSC-A
Intact cells
SSC
FSC
Remaining neutrophils (17885)
Ly6g
Remaining healthy neutrophils (11254)
PI
AV
c
Remaining neutrophil number (x10^4)
WT
Gsdmd KO
ns
0h 8h 12h 16h 20h
AV-PI- (healthy) neutrophil number (x10^4)
AV+PI+ neutrophil number (x10^4)
AV+PI- neutrophil number (x10^4)
d
Cell number(x10^4)
Apoptotic cell
Lytic cell
Healthy cell
e
The ratio of apoptosis to lytic cell death
f
Lytic cell death
Remain healthy
Apoptosis
WT
Gsdmd KO
Neutrophil aging and death

Fig.S7. GSDMD deficiency delayed neutrophil death programs but did not alter the mode of neutrophil death.

(a) GSDMD cleavage during programed spontaneous neutrophil death. Neutrophils from WT mouse bone marrow were cultured for indicated time periods. β-actin was used as a protein loading control. Results are representative of three biological replicates. Relative levels of cleaved GSDMD-NT were quantified based on densitometry. Results are means (±SD) of three independent experiments. Cleavage of GSDMD in WT neutrophils treated with LPS for 3 hours followed by nigericin for 3 hours served as a positive control. Cleavage of GSDMD in *Gsdmd* KO neutrophils cultured for 8 hours was used as the negative control.

(b) Representative flow cytometric pictures showing gating strategy for assessing neutrophil survival rate using counting beads. Purified WT neutrophils were cultured for 20 h and stained with Annexin V and PI. Results are representative of at least 3 independent experiments.

(c) The absolute number of remaining intact, healthy (PI^-AV^-), apoptotic (PI^-AV^+), and PI^+AV^+ neutrophils at each time point assessed by flow cytometry using counting beads. The measurement was performed as described in **(b)**. All data are represented as mean ± SD. n = 4 independent repeats, *P < 0.05, **P < 0.01. ns, not significant (P > 0.05).

(g) (d) The absolute number of healthy neutrophils and neutrophils undergoing apoptosis or lytic cell death at the indicated time points. The absolute number of each cell population was calculated as the product of the initial cell count (time 0) times its percentage at each time point. To calculate the percentages, at least 200 cells were tracked. All data are represented as mean (n=4 independent repeats).

(e) The ratio of neutrophils undergoing apoptosis to neutrophils undergoing lytic cell death at the indicated time points. All data are represented as mean ± SD. ns, not significant (P > 0.05).

(f) Summary of programmed spontaneous death of WT and *Gsdmd* KO neutrophils.

Source data are provided as a Source Data file.

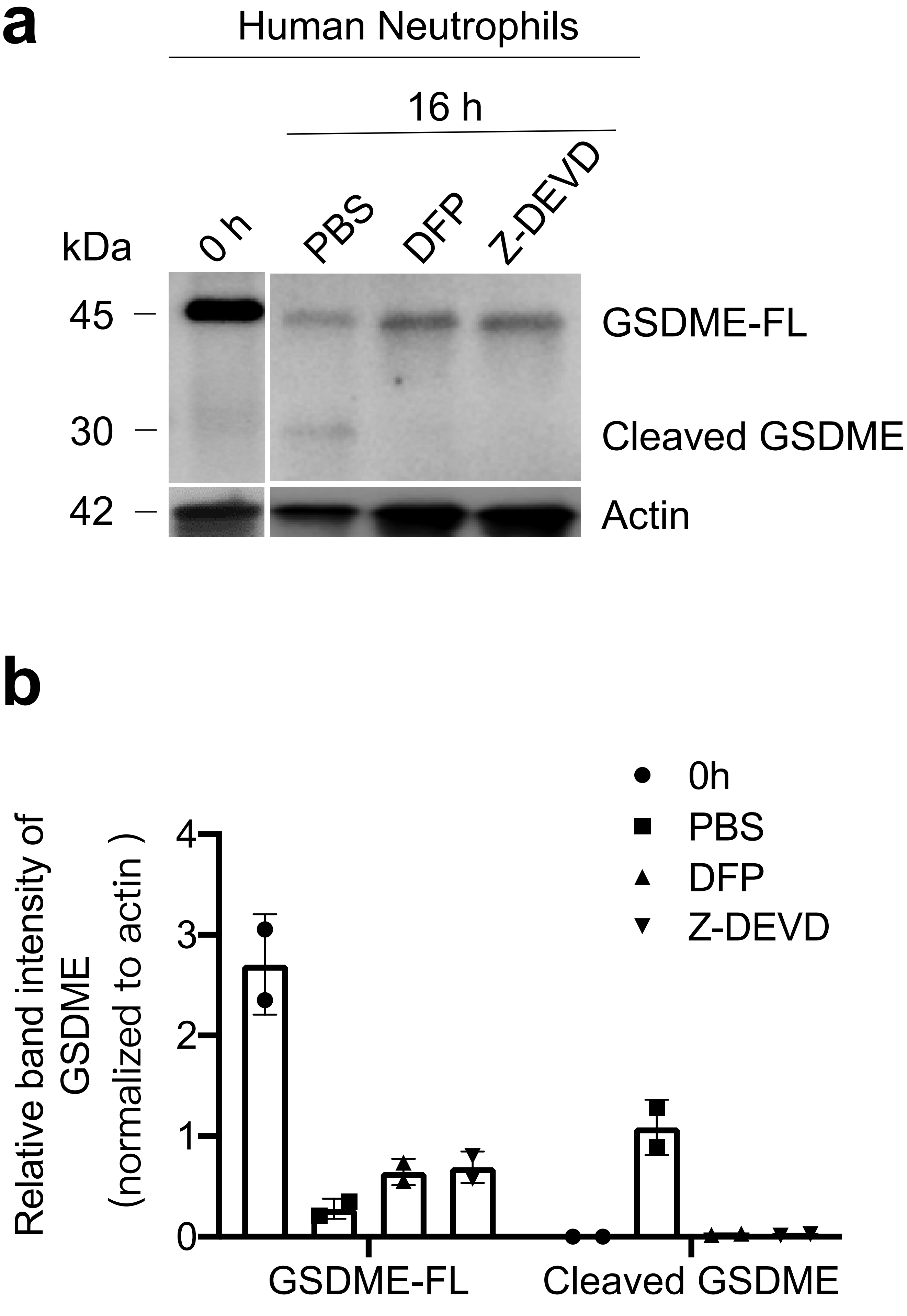
a
Human Neutrophils
16 h
kDa
0 h
PBS
DFP
Z-DEVD
45
30
42
GSDME-FL
Cleaved GSDME
Actin
b
0h
PBS
DFP
Z-DEVD
Relative band intensity of GSDME (normalized to actin)
0
1
2
3
4
GSDME-FL
Cleaved GSDME

Fig.S8. Cleavage of GSDME during the death of human neutrophils.

(a) Human neutrophils were isolated from healthy donors and cultured in the presence or absence of DFP (100 µM) or Z-DEVD-FMK (100 µM) for 16 hours. After incubation, the cells were lysed using 1X RIPA Lysis buffer containing DFP (100 µM), PMSF (0.1 mM), and the Halt proteinase inhibitor cocktail (1x). This mixture was then boiled for 5 minutes. Western blot analyses were conducted using an anti-DFNA5 polyclonal antibody (Sigma-Aldrich, HPA011326, 1:1000) in a 5% non-fat dry milk solution. Image capture was done with a Biorad-chemidoc machine. β-actin served as a protein loading control. The results presented are representative of at least three biological replicates. Of note, the size of the full-length GSDMD in neutrophils was smaller than that detected in other cell types using the same antibody, suggesting an alternatively spliced form in neutrophils.

(b) Protein levels were quantified through densitometry. The results, represented as means (±SD), are from three independent experiments. **$P < 0.01$ when compared to PBS-treated neutrophils, as determined by the Student's t-test.

Source data are provided as a Source Data file.

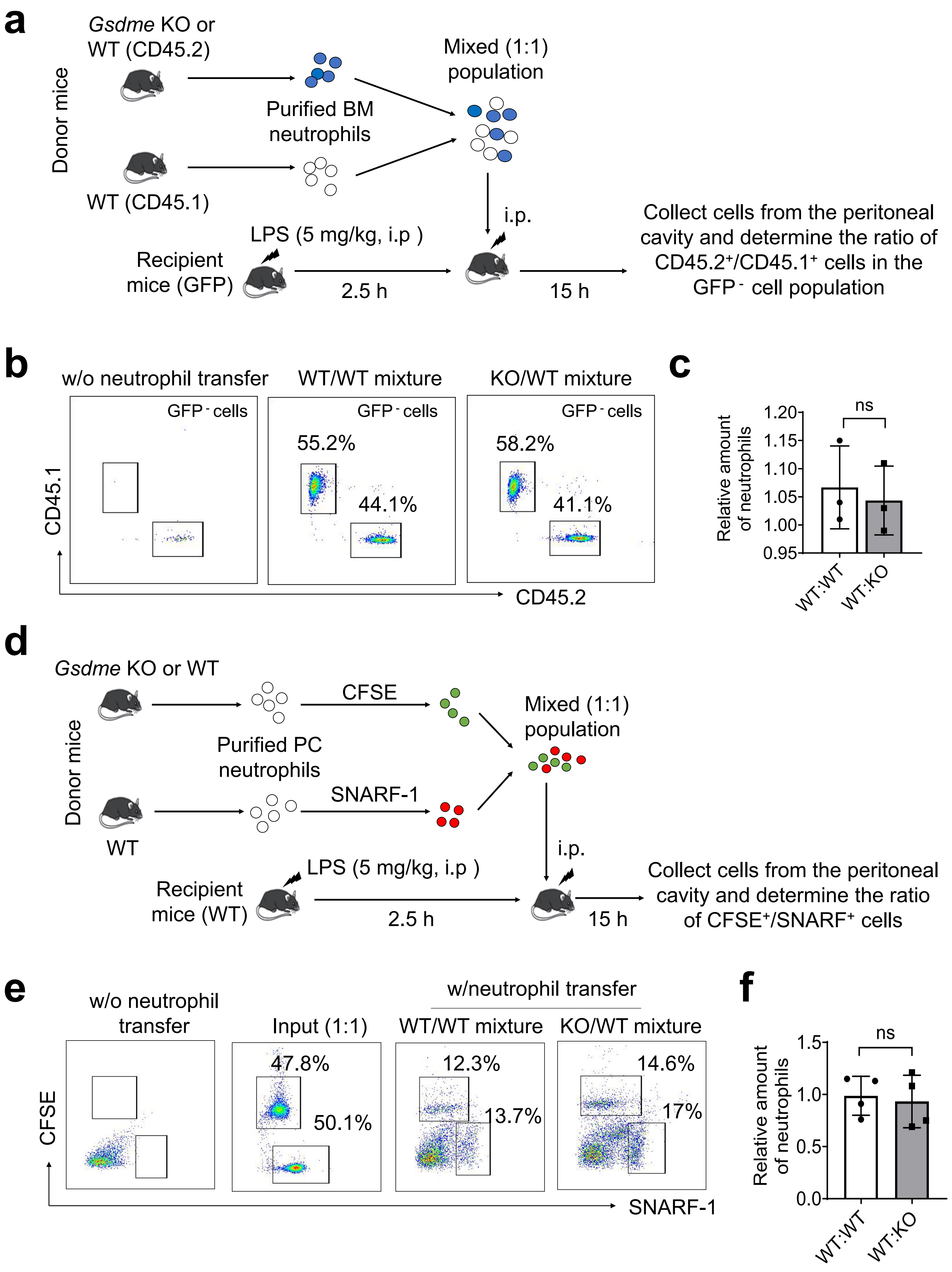
a
Gsdme KO or WT (CD45.2)
Donor mice
Purified BM neutrophils
Mixed (1:1) population
WT (CD45.1)
i.p.
LPS (5 mg/kg, i.p)
Recipient mice (GFP)
2.5 h
15 h
Collect cells from the peritoneal cavity and determine the ratio of CD45.2⁺/CD45.1⁺ cells in the GFP⁻ cell population
b
w/o neutrophil transfer
WT/WT mixture
KO/WT mixture
GFP⁻ cells
55.2%
44.1%
58.2%
41.1%
CD45.1
CD45.2
c
Relative amount of neutrophils
ns
1.20
1.15
1.10
1.05
1.00
0.95
WT:WT
WT:KO
d
Gsdme KO or WT
Donor mice
Purified PC neutrophils
CFSE
SNARF-1
Mixed (1:1) population
WT
i.p.
LPS (5 mg/kg, i.p)
Recipient mice (WT)
2.5 h
15 h
Collect cells from the peritoneal cavity and determine the ratio of CFSE⁺/SNARF⁺ cells
e
w/o neutrophil transfer
Input (1:1)
w/neutrophil transfer
WT/WT mixture
KO/WT mixture
47.8%
50.1%
12.3%
13.7%
14.6%
17%
CFSE
SNARF-1
f
Relative amount of neutrophils
ns
1.5
1.0
0.5
0.0
WT:WT
WT:KO

Fig.S9. GSDME disruption in neutrophils did not affect overall neutrophil survival *in vivo*.

(a) Experimental scheme for assessing the relative *in vivo* survival of WT and *Gsdme* KO neutrophils in a mouse peritonitis model. GFP positive mice were used as recipient mice to distinguish neutrophils from donor mice and recipient mice.

(b) Representative flow cytometric pictures of transferred neutrophil mixtures. Cells in peritoneal lavage fluid were stained with PE-CD45.1, APC-CD45.2, and BV421-Ly6g and were analyzed by flow cytometry. Results are representative of at least three biological replicates.

(c) The relative amount of $CD45.1^+$ (WT) and $CD45.2^+$ (*Gsdme* KO) neutrophils in the GFP^- population. Shown is a representative result from one of three independent experiments. All data are represented as mean ± SD. n = 3 mice, ns, non-significant.

(d) Experimental scheme for assessing the relative *in vivo* survival of WT and *Gsdme* KO neutrophils based on SNARF1 and CFSE staining.

(e) Representative flow cytometric pictures of transferred neutrophil mixtures. Results are representative of at least three biological replicates.

(f) The relative amount of transferred WT and GSDME-deficient neutrophils. The input control was normalized to 1. Shown is a representative result from one of three independent experiments. All data are represented as mean ± SD. n=4 mice, ns, non-significant.

Source data are provided as a Source Data file.

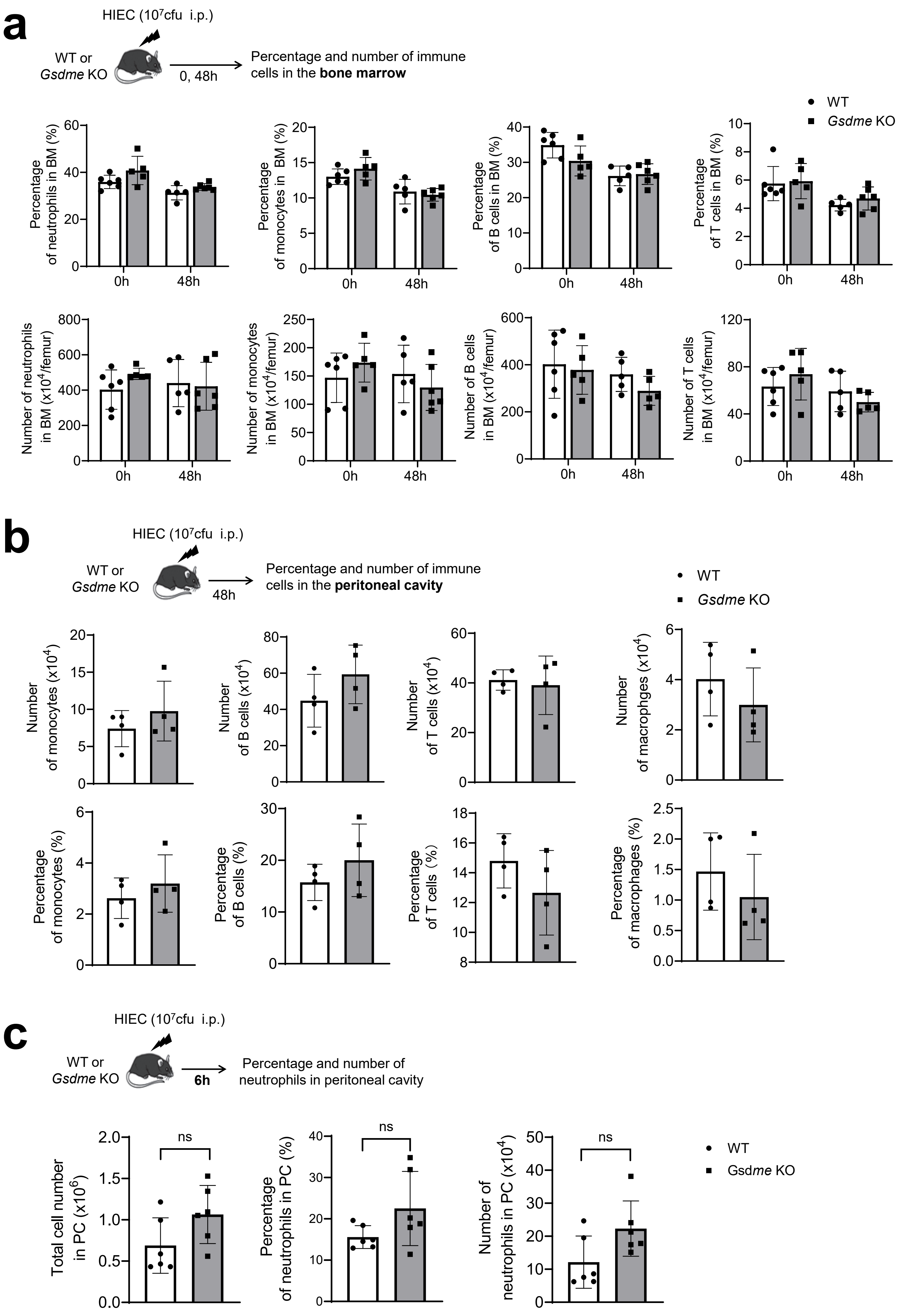

a
HIEC (10^7cfu i.p.)
WT or Gsdme KO
0, 48h
Percentage and number of immune cells in the bone marrow
WT
Gsdme KO
Percentage of neutrophils in BM (%)
Percentage of monocytes in BM (%)
Percentage of B cells in BM (%)
Percentage of T cells in BM (%)
Number of neutrophils in BM (x10^4/femur)
Number of monocytes in BM (x10^4/femur)
Number of B cells in BM (x10^4/femur)
Number of T cells in BM (x10^4/femur)
0h
48h
b
HIEC (10^7cfu i.p.)
WT or Gsdme KO
48h
Percentage and number of immune cells in the peritoneal cavity
WT
Gsdme KO
Number of monocytes (x10^4)
Number of B cells (x10^4)
Number of T cells (x10^4)
Number of macrophges (x10^4)
Percentage of monocytes (%)
Percentage of B cells (%)
Percentage of T cells (%)
Percentage of macrophages (%)
c
HIEC (10^7cfu i.p.)
WT or Gsdme KO
6h
Percentage and number of neutrophils in peritoneal cavity
Total cell number in PC (x10^6)
Percentage of neutrophils in PC (%)
Number of neutrophils in PC (x10^4)
ns
WT
Gsdme KO

Fig.S10. Impact of whole-body GSDME knockout on immune cell numbers and trafficking to inflammatory sites.

(a) GSDME disruption did not alter immune cell count in the BM. Shown are the percentage and number of neutrophils, monocytes, B cells, and T cells in the bone marrow of unchallenged and HIEC-challenged WT and *Gsdme* KO mice. All data are represented as mean ± SD, n=5-6 mice.

(b) GSDME disruption did not alter the percentage and number of monocytes, B cells, T cells, and macrophages in inflamed peritoneal cavities. All data are represented as mean ± SD, n=4 mice.

(c) GSDME disruption did not affect neutrophil recruitment to the peritoneal cavity in peritonitis. Neutrophil recruitment was assessed 6 h after HIEC injection. All data are represented as mean ± SD, n = 6 mice. ns, not significant (P > 0.05).

Source data are provided as a Source Data file.

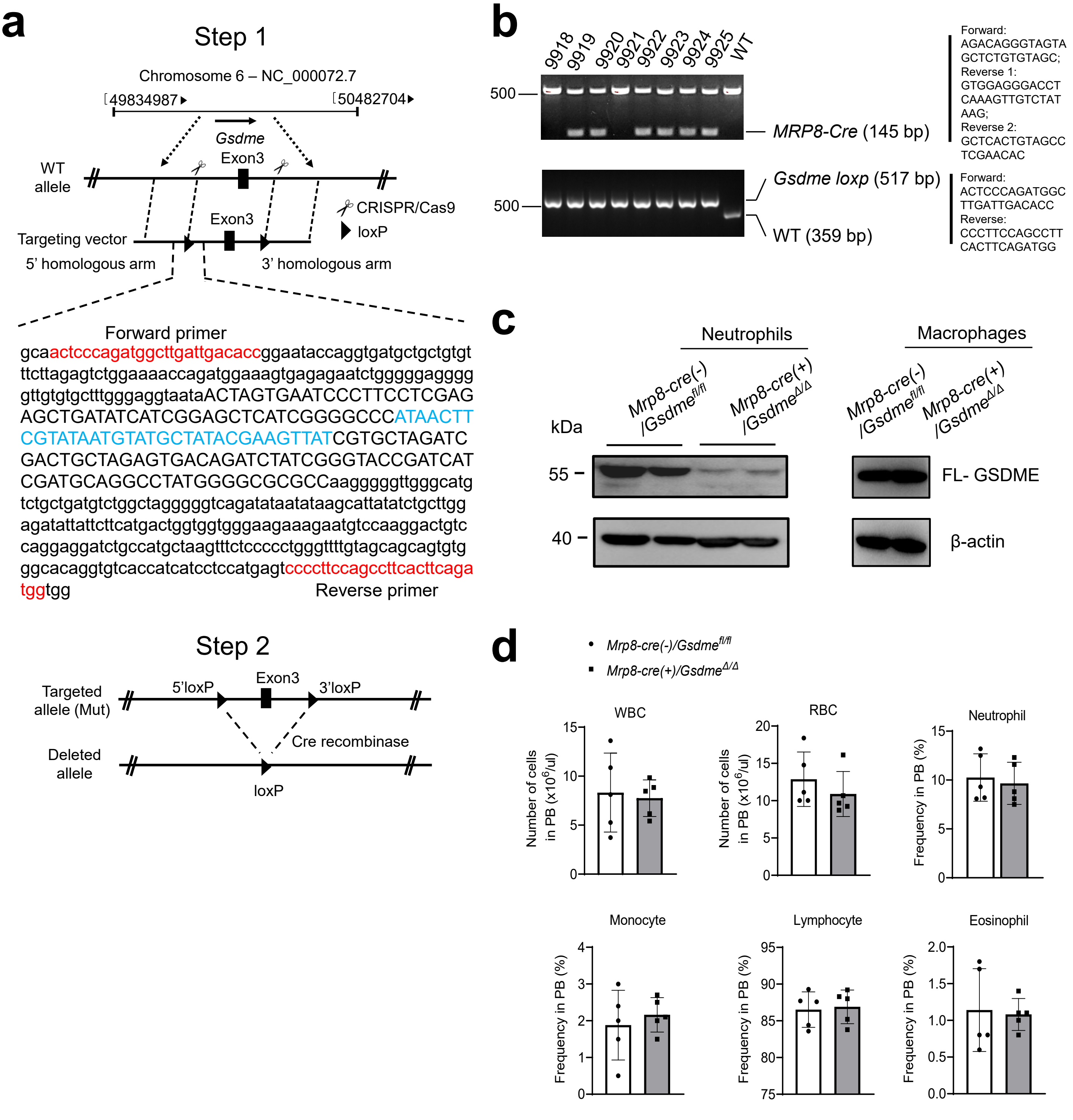
a
Step 1
Chromosome 6 – NC_000072.7
49834987
50482704
Gsdme
Exon3
WT allele
CRISPR/Cas9
loxP
Exon3
Targeting vector
5' homologous arm
3' homologous arm
Forward primer
gcaactcccagatggcttgattgacaccggaataccaggtgatgctgctgtgt
ttcttagagtctggaaaaccagatggaaagtgagagaatctgggggagggg
gttgtgtgctttgggaggtaataACTAGTGAATCCCTTCCTCGAG
AGCTGATATCATCGGAGCTCATCGGGGCCCATAACTT
CGTATAATGTATGCTATACGAAGTTATCGTGCTAGATC
GACTGCTAGAGTGACAGATCTATCGGGTACCGATCAT
CGATGCAGGCCTATGGGGCGCGCCaagggggttgggcatg
tctgctgatgtctggctagggggtcagatataatataagcattatatctgcttgg
agatattattcttcatgactggtggtgggaagaaagaatgtccaaggactgtc
caggaggatctgccatgctaagtttctcccctgggttttgtagcagcagtgtg
ggcacaggtgtcaccatcatcctccatgagtcccttccagccttcacttcaga
tggtgg
Reverse primer
Step 2
Targeted allele (Mut)
5'loxP
Exon3
3'loxP
Cre recombinase
Deleted allele
loxP
b
9918 9919 9920 9921 9922 9923 9924 9925 WT
500
MRP8-Cre (145 bp)
Forward: AGACAGGGTAGTAGCTCTGTGTAGC;
Reverse 1: GTGGAGGGACCTCAAAGTTGTCTATAAG;
Reverse 2: GCTCACTGTAGCCTCGAACAC
500
Gsdme loxp (517 bp)
WT (359 bp)
Forward: ACTCCCAGATGGCTTGATTGACACC
Reverse: CCCTTCCAGCCTTCACTTCAGATGG
c
Neutrophils
Macrophages
kDa
Mrp8-cre(-)/Gsdme fl/fl
Mrp8-cre(+)/Gsdme Δ/Δ
Mrp8-cre(-)/Gsdme fl/fl
Mrp8-cre(+)/Gsdme Δ/Δ
55
40
FL- GSDME
β-actin
d
Mrp8-cre(-)/Gsdme fl/fl
Mrp8-cre(+)/Gsdme Δ/Δ
WBC
Number of cells in PB (x10^6/ul)
RBC
Number of cells in PB (x10^6/ul)
Neutrophil
Frequency in PB (%)
Monocyte
Frequency in PB (%)
Lymphocyte
Frequency in PB (%)
Eosinophil
Frequency in PB (%)

Fig.S11. Generation of neutrophil-specific *Gsdme* conditional KO mice (*Mrp8-cre(+)/Gsdme*$^{\Delta/\Delta}$).

(a) Schematic outline for the generation of neutrophil-specific *Gsdme* conditional KO mice (*Mrp8-cre(+)/Gsdme*$^{\Delta/\Delta}$). *Mrp8-cre(+)/Gsdme*$^{\Delta/\Delta}$ were generated by crossing *Gsdme* loxP-flanked mice with *B6.Cg-Tg(S100A8-cre,-EGFP)1Ilw/J* mice. Heterozygous F1 parents were mated to generate *Mrp8-cre(-)/Gsdme*$^{fl/fl}$ (WT controls) and *Mrp8-cre(+)/Gsdme*$^{\Delta/\Delta}$ littermates. Forward primer, loxP site (blue), and reverse primer sequences are shown.

(b) PCR result showing DNA bands representing *MRP-Cre*, *Gsdme loxP*, and WT in indicated mice. Related primer sequences are shown.

(c) Neutrophil-specific deletion of *Gsdme* was confirmed by western blotting. Bone marrow-derived neutrophils (highly purified) and macrophages from *Mrp8-cre(-)/Gsdme*$^{fl/fl}$ and *Mrp8-cre(+)/Gsdme*$^{\Delta/\Delta}$ mice were used. Results are representative of at least three independent experiments.

(d) Differential blood counts showing absolute numbers of WBCs and RBCs and the relative frequencies of different leukocytes in the peripheral blood of *Mrp8-cre(-)/Gsdme*$^{fl/fl}$ and *Mrp8-cre(+)/Gsdme*$^{\Delta/\Delta}$ mice. Data represented as mean ± SD, n=5 mice/group.

Source data are provided as a Source Data file.

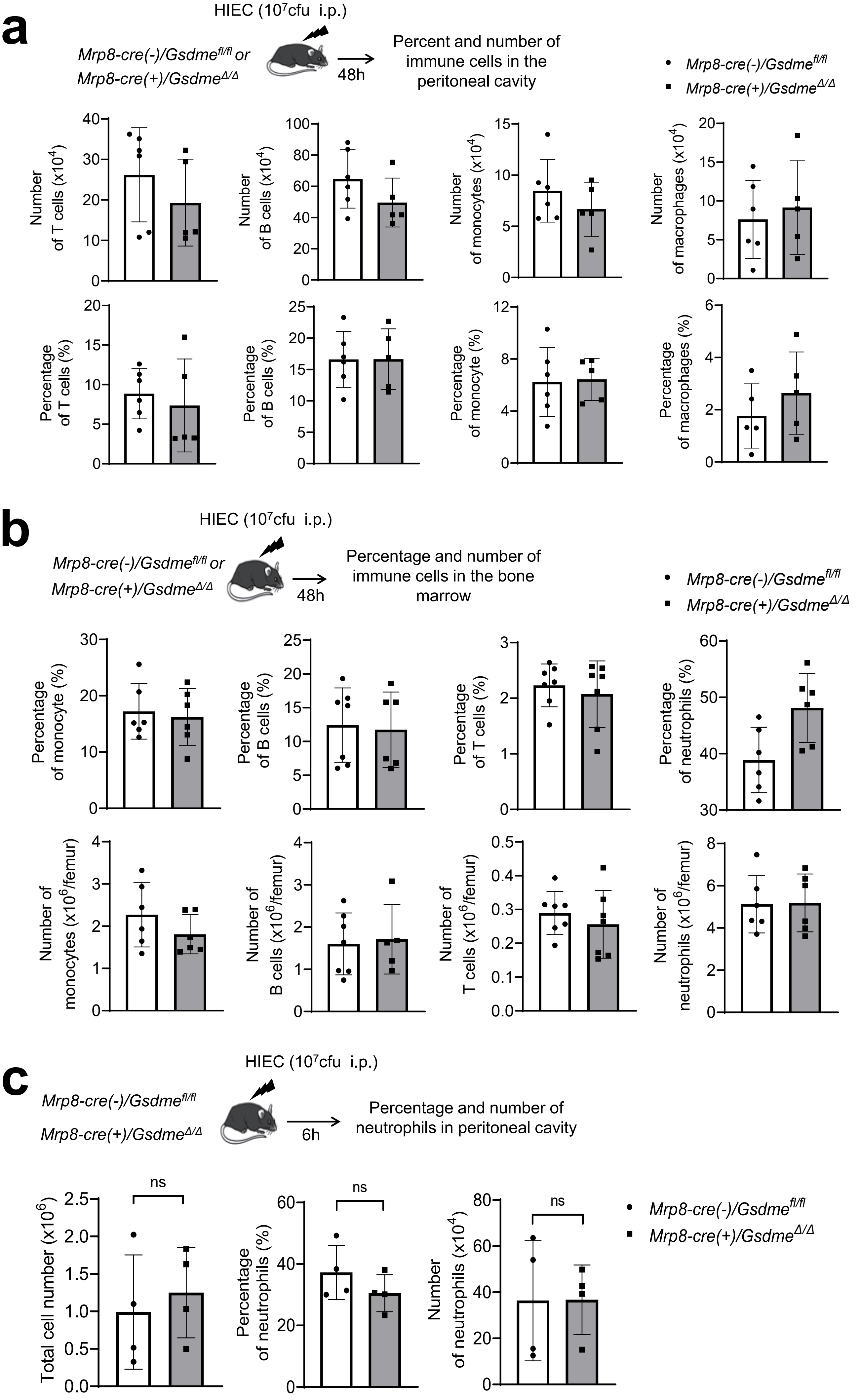

a
HIEC (10^7cfu i.p.)
Mrp8-cre(-)/Gsdme$^{fl/fl}$ or
Mrp8-cre(+)/Gsdme$^{Δ/Δ}$
48h
Percent and number of immune cells in the peritoneal cavity
Mrp8-cre(-)/Gsdme$^{fl/fl}$
Mrp8-cre(+)/Gsdme$^{Δ/Δ}$
Number of T cells ($x10^4$)
Number of B cells ($x10^4$)
Number of monocytes ($x10^4$)
Number of macrophages ($x10^4$)
Percentage of T cells (%)
Percentage of B cells (%)
Percentage of monocyte (%)
Percentage of macrophages (%)
b
HIEC (10^7cfu i.p.)
Mrp8-cre(-)/Gsdme$^{fl/fl}$ or
Mrp8-cre(+)/Gsdme$^{Δ/Δ}$
48h
Percentage and number of immune cells in the bone marrow
Mrp8-cre(-)/Gsdme$^{fl/fl}$
Mrp8-cre(+)/Gsdme$^{Δ/Δ}$
Percentage of monocyte (%)
Percentage of B cells (%)
Percentage of T cells (%)
Percentage of neutrophils (%)
Number of monocytes ($x10^6$/femur)
Number of B cells ($x10^6$/femur)
Number of T cells ($x10^6$/femur)
Number of neutrophils ($x10^6$/femur)
c
HIEC (10^7cfu i.p.)
Mrp8-cre(-)/Gsdme$^{fl/fl}$
Mrp8-cre(+)/Gsdme$^{Δ/Δ}$
6h
Percentage and number of neutrophils in peritoneal cavity
Total cell number ($x10^6$)
Percentage of neutrophils (%)
Number of neutrophils ($x10^4$)
ns
Mrp8-cre(-)/Gsdme$^{fl/fl}$
Mrp8-cre(+)/Gsdme$^{Δ/Δ}$

Fig.S12. Impact of neutrophil-specific GSDME knockout on immune cell numbers and trafficking to inflammatory sites.

(a) GSDME disruption in neutrophils did not alter the percentage and number of monocytes, B cells, T cells, and macrophages in inflamed peritoneal cavities. All data are represented as mean ± SD, n=5-6 mice.

(b) GSDME disruption in neutrophils did not alter immune cell count in the BM of HIEC-challenged mice. Shown are percentage and number of neutrophils, monocytes, B cells, and T cells in the bone marrow of HIEC-challenged *Mrp8-cre(-)/Gsdme*$^{fl/fl}$ and *Mrp8-cre(+)/Gsdme*$^{\Delta/\Delta}$ mice. All data are represented as mean ± SD, n=6 mice.

(c) GSDME disruption in neutrophils did not affect neutrophil recruitment to the peritoneal cavity in peritonitis. Neutrophil recruitment was assessed 6 h after HIEC injection. All data are represented as mean ± SD, n = 4 mice. ns, not significant (P > 0.05).

Source data are provided as a Source Data file.

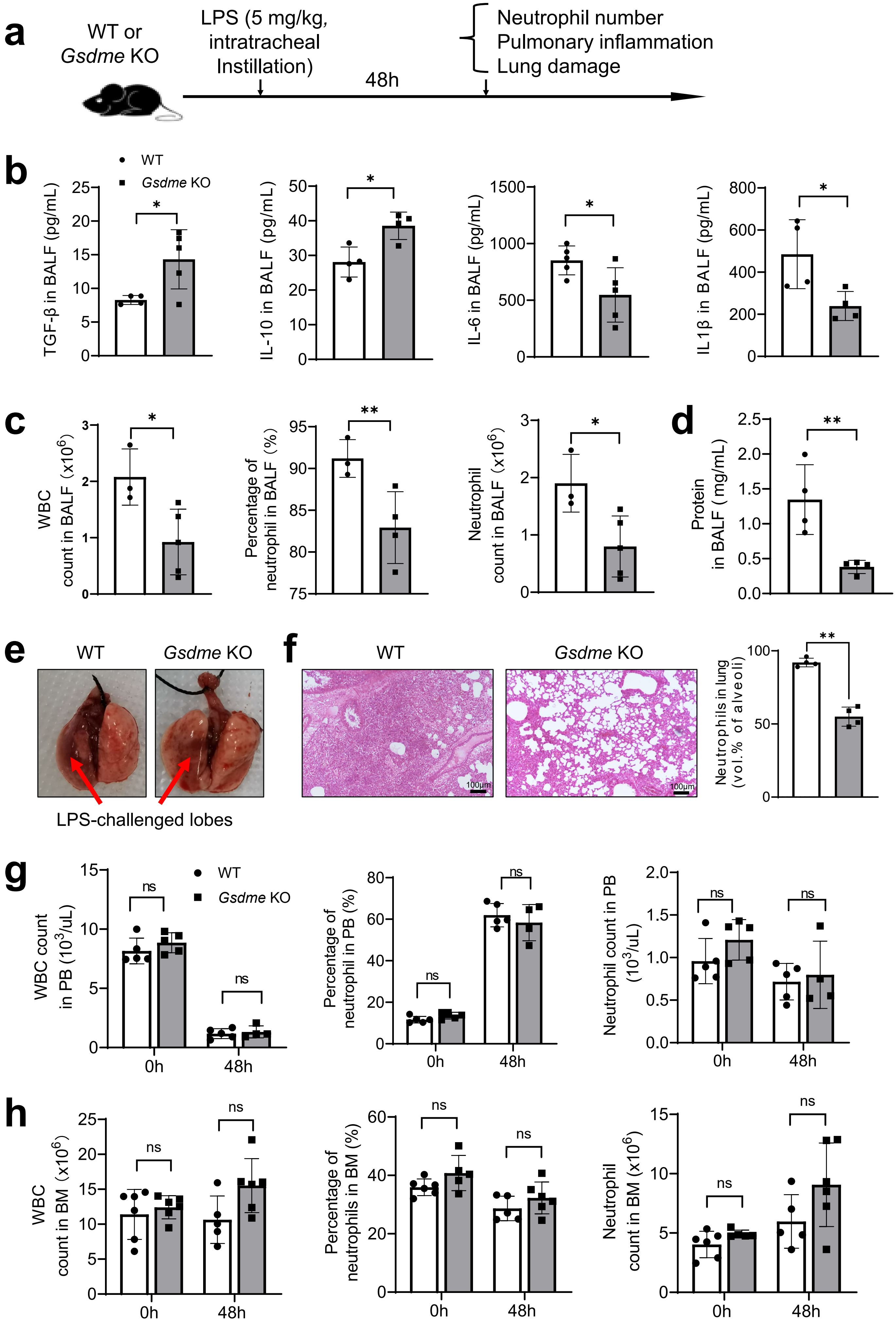
a
WT or Gsdme KO
LPS (5 mg/kg, intratracheal Instillation)
48h
Neutrophil number
Pulmonary inflammation
Lung damage
b
WT
Gsdme KO
TGF-β in BALF (pg/mL)
IL-10 in BALF (pg/mL)
IL-6 in BALF (pg/mL)
IL1β in BALF (pg/mL)
c
WBC count in BALF (x10^6)
Percentage of neutrophil in BALF (%)
Neutrophil count in BALF (x10^6)
d
Protein in BALF (mg/mL)
e
WT
Gsdme KO
LPS-challenged lobes
f
WT
Gsdme KO
Neutrophils in lung (vol.% of alveoli)
g
WT
Gsdme KO
WBC count in PB (10^3/uL)
Percentage of neutrophil in PB (%)
Neutrophil count in PB (10^3/uL)
0h
48h
h
WBC count in BM (x10^6)
Percentage of neutrophils in BM (%)
Neutrophil count in BM (x10^6)
0h
48h

Fig.S13. GSDME disruption attenuated inflammation and alleviated lung injury during LPS-induced pneumonia.

(a) Experimental scheme for assessing LPS-induced pulmonary inflammation and associated lung injury in WT and whole-body *Gsdme* KO mice. The experiment was conducted as described in **Fig.5**. Mice were challenged with LPS (5 mg/kg body weight) and sacrificed after 48 hours.

(b) Levels of indicated cytokines in BALF of WT and *Gsdme* KO mice were determined by enzyme-linked immunosorbent assay (ELISA). All data are represented as mean ± SD, n ≥ 4 mice, *P < 0.05.

(c) The number and percentage of neutrophils in BALF. The total number of cells in the BALF was counted with a hemocytometer. Differential cell counts were determined by FACS analysis. Cells were stained with APC-CD11b and PE-Ly6g. Neutrophils were identified as $Ly6g^{+}CD11b^{+}$ cells. Neutrophil count in BALF was calculated as follows: neutrophil count in BALF = total cell count in BALF × % PMN. All data are represented as mean ± SD, n ≥ 3 mice, *P < 0.05, **P < 0.01.

(d) BALF total protein levels. Protein accumulation in the inflamed lung was measured using a protein assay kit. All data are represented as mean ± SD, n = 4 mice, **P < 0.01.

(e) Representative images of the lungs of LPS-challenged WT and *Gsdme* KO mice. The lung lobes challenged with LPS are indicated. Results are representative of at least three biological replicates.

(f) Representative hematoxylin and eosin (H&E)-stained images of LPS-challenged lung tissues. Results are representative of at least three biological replicates. Neutrophil recruitment to alveoli was quantified as volume fraction of the alveolar spaces. All data are represented as mean ± SD, n = 4 mice, **P < 0.01.

(g) The number and percentage of neutrophils in the peripheral blood (PB) of WT and *Gsdme* KO mice before (0 h) and after (48 h) LPS challenge. The PB WBC and differential cell counts were measured by an automated hematology analyzer. All data are represented as mean ± SD. n ≥ 4 mice; ns, no significant difference (P > 0.05).

(h) The number and percentage of neutrophils in the bone marrow (BM) of WT and *Gsdme* KO mice before (0 h) and after (48 h) LPS challenge. The total number of cells in the bone marrow was counted with a hemocytometer. Differential cell counts were determined by FACS analysis. Neutrophil count in BM was calculated as the product of the total cell count in the BM times the percentage of neutrophils. Data are represented as mean ± SD. n ≥ 5 mice in each group. ns, no significant difference (P > 0.05).

Source data are provided as a Source Data file.

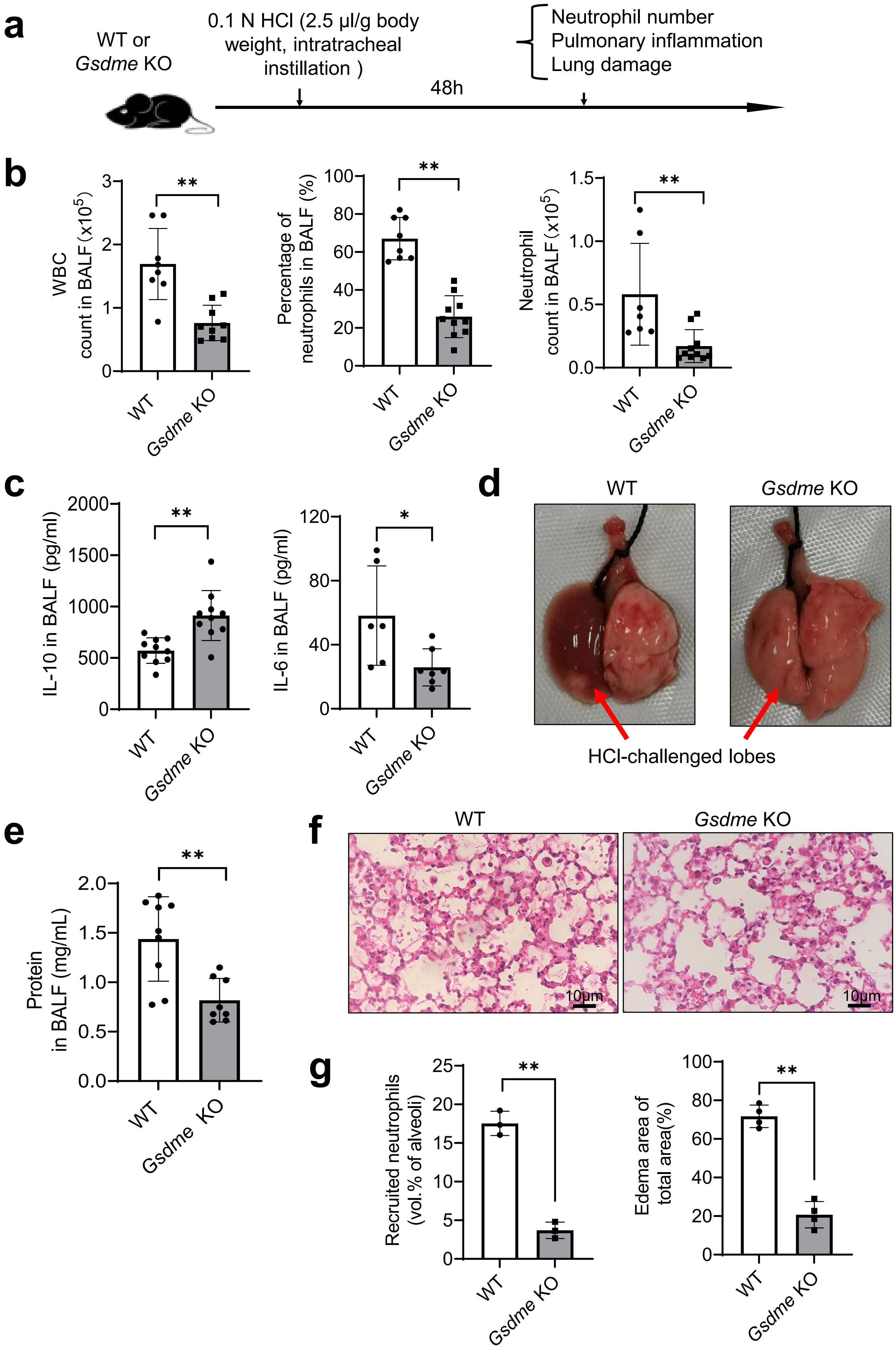
a
WT or Gsdme KO
0.1 N HCl (2.5 μl/g body weight, intratracheal instillation)
48h
Neutrophil number
Pulmonary inflammation
Lung damage
b
WBC count in BALF (x10^5)
Percentage of neutrophils in BALF (%)
Neutrophil count in BALF (x10^5)
WT
Gsdme KO
c
IL-10 in BALF (pg/ml)
IL-6 in BALF (pg/ml)
d
WT
Gsdme KO
HCl-challenged lobes
e
Protein in BALF (mg/mL)
f
WT
Gsdme KO
10μm
g
Recruited neutrophils (vol.% of alveoli)
Edema area of total area(%)

Fig.S14. GSDME disruption attenuated inflammation and alleviated lung injury in acid aspiration pneumonitis.

(a) Experimental scheme for assessing acid-induced pulmonary inflammation and associated lung injury in WT and whole-body *Gsdme* KO mice. Mice were challenged with 0.1 N HCl (2.5 μl/g body weight) and sacrificed after 48 hours.

(b) The number and percentage of neutrophils in BALF. WBC and neutrophil counts were determined by FACS analysis using counting beads as described in **Fig.S3**. Cells were stained with APC-CD11b and PE-Ly6g antibodies. Neutrophils were identified as $Ly6g^+CD11b^+$ cells. All data are represented as mean ± SD, n ≥ 7 mice, **P < 0.01.

(c) IL-10 and IL-6 levels in the BALF of WT and *Gsdme* KO mice were determined by ELISA. All data are represented as mean ± SD, n ≥ 6 mice, *P < 0.05, **P < 0.01.

(d) Representative images of the lungs of acid-challenged WT and *Gsdme* KO mice. The lung lobes challenged with HCl are indicated. Results are representative of at least three biological replicates.

(e) BALF total protein levels. Protein accumulation in the inflamed lung was measured using a protein assay kit. All data are represented as mean ± SD, n ≥ 8 mice, **P < 0.01.

(f) Representative hematoxylin and eosin (H&E)-stained images of acid-challenged lung tissues. The scale bar is 10 μm. Results are representative of at least three biological replicates.

(g) Neutrophil recruitment to alveoli was quantified as volume fraction of the alveolar spaces occupied by neutrophils. Pulmonary edema formation was quantified as the percentage of edema area in the total parenchymal region. Data are represented as mean ± SD of three experiments. n ≥ 4 mice in each group. *P < 0.01.

Source data are provided as a Source Data file.

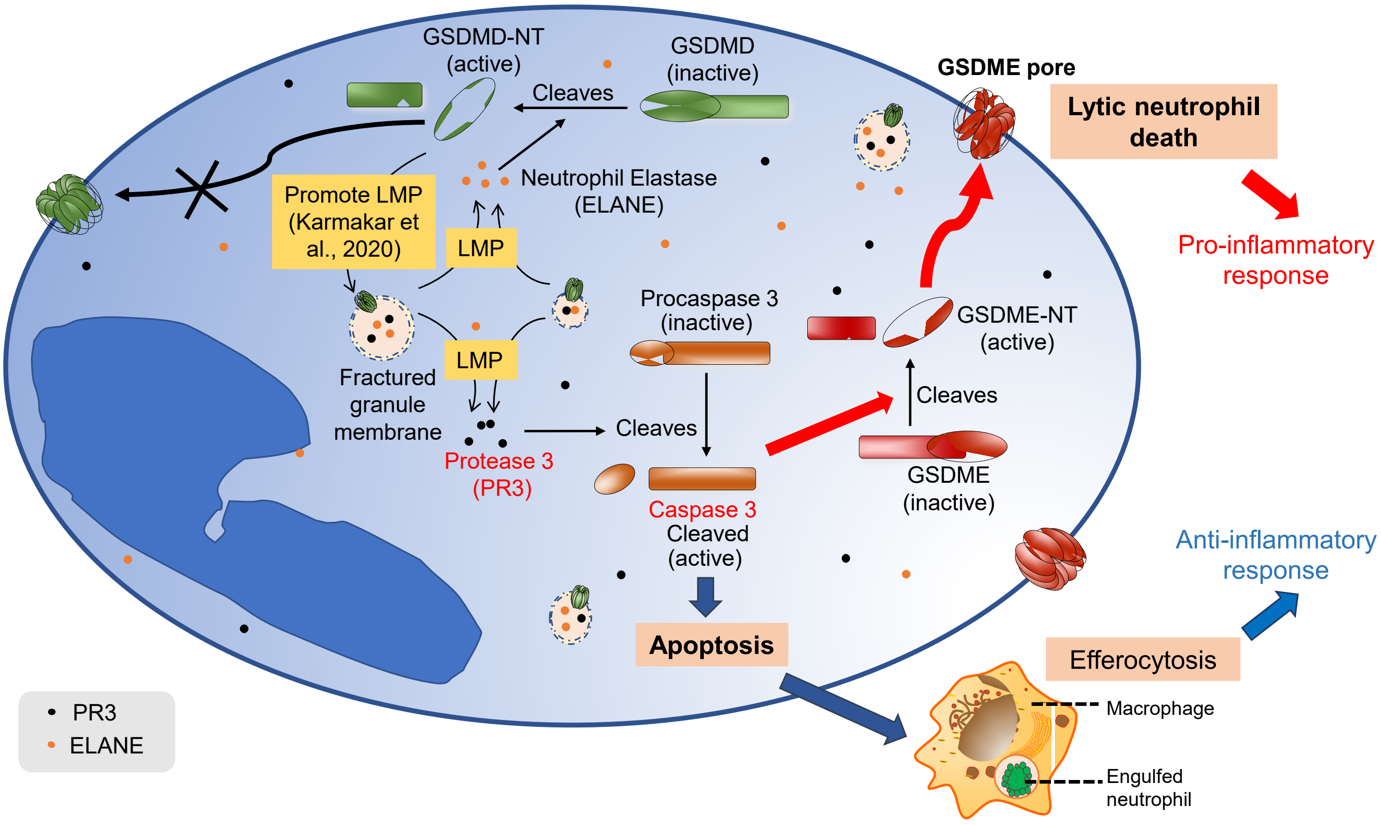

GSDMD-NT
(active)
GSDMD
(inactive)
Cleaves
GSDME pore
Lytic neutrophil death
Neutrophil Elastase
(ELANE)
Promote LMP
(Karmakar et al., 2020)
LMP
Pro-inflammatory response
Procaspase 3
(inactive)
GSDME-NT
(active)
LMP
Fractured
granule
membrane
Cleaves
Cleaves
Protease 3
(PR3)
Caspase 3
Cleaved
(active)
GSDME
(inactive)
Anti-inflammatory response
Apoptosis
Efferocytosis
Macrophage
Engulfed
neutrophil
PR3
ELANE

Fig.S15. GSDMD and GSDME exert different functions in neutrophil spontaneous death. In neutrophils, although N-GSDMD is required for IL-1β secretion, it does not form pores on the plasma membrane and thus does not directly induce cell permeability or pyroptosis[2]. Instead, it is predominantly localized on and permeabilizes azurophilic granule membranes, amplifying lysosomal membrane permeabilization (LMP) and further promoting leakage of neutrophil serine proteases into the cytosol, with PR3 mediating caspase-3 activation and ELANE mediating secondary cleavage of GSDMD. Thus, GSDMD contributes to both apoptosis and pyroptosis. Its disruption leads to an overall increase in healthy neutrophil number but does not alter the mode of neutrophil death. In contrast, GSDME acts downstream of caspase-3 and only contributes to pyroptosis without affecting the level of caspase activation. GSDME dictates the mode of neutrophil death. Its disruption skews neutrophil death to apoptosis but does not alter the number of healthy neutrophils.

Supplemental Methods

Confirmation of neutrophil-specific *Gsdme* deletion in the conditional *Gsdme* KO mice (*Mrp8-cre(+)/Gsdme*$^{\Delta/\Delta}$)

Standard genotyping and western blotting were used to confirm neutrophil-specific deletion of *Gsdme*.

Following primers were used for genotyping:

Gsdme flox:

5'loxP-F: ACTCCCAGATGGCTTGATTGACACC

5'loxP-R: CCATCTGAAGTGAAGGCTGGAAGGG

3'loxP-F: GCACCTACTATGTGTCAGGCAGTGG

3'loxP-R: CCTTAGCATGCTCCTCGTGTCTCAG

Mrp8-Cre:

Forward: AGACAGGGTAGTAGCTCTGTGTAGC

Reverse 1: GTGGAGGGACCTCAAAGTTGTCTATAAG

Reverse 2: GCTCACTGTAGCCTCGAACAC

Hematology and flow cytometry

Blood samples (20 µL) were taken from the retroorbital sinuses of anesthetized mice using heparinized capillary tubes (Modulohm). Peripheral blood was assayed with an automatic hematology analyzer (Mindray, Shenzhen, China). BM and BALF cells were collected and then centrifuged at 400 x g for 5 min. Total and differential cell counts were determined from the pelleted cell fraction by flow cytometry. For flow cytometry, cells were suspended in 200 µL ice-cold PBS, blocked with TruStain FcX™ (anti-mouse CD16/32, BioLegend, #101320, Clone 93) at 4°C for 20 min, and stained with PE/Cyanine7-CD45 (BioLegend, # 103114, Clone 30-F11) APC/Cyanine7-F4/80 (BioLegend, #123118, Clone BM8), APC-CD11b (BioLegend, #101212,Clone M1/70), PE-Ly6g (Biolegend, #127608, Clone 1A8), and FITC-Ly6c antibodies (BioLegend, #128006,Clone HK1.4) at 4°C for 20 min. Flow cytometry was conducted using a BD Canto II flow cytometer, and the results were analyzed using FlowJo software (BD Biosciences, Franklin Lakes, NJ).

Murine bone marrow neutrophil isolation

Murine bone marrow neutrophils were isolated as previously described[3]. Briefly, bone marrow from the femurs and tibias of 8-12 weeks old male WT and *Gsdme* KO mice was flushed out with 5 ml of Hanks' balanced salt solution (HBSS)/EDTA/bovine serum albumin (BSA). Cells were spun down (400 x g, 5 min, RT), resuspended in 1 ml of HBSS/EDTA/BSA, layered over discontinuous Percoll/HBSS gradients (52, 62, and 76%), and centrifuged (1060 x g, 30 min, RT). The interface between the 62% and 76% layers containing neutrophils was

harvested and washed with 5 ml of HBSS/EDTA/BSA. To remove RBCs, the cell suspension was overlayed on top of Histopaque-1119 (Sigma-Aldrich, St Louis, MO) and centrifuged (1600 x g) for 30 min at RT. The interface between the cell suspension and Histopaque-1119 layers containing neutrophils was harvested and washed with 5 ml of HBSS/EDTA/BSA, and cells were spun down (500 x g, 6 min, RT) and resuspended in the desired medium or buffer. Neutrophils preps with >85% purity were routinely obtained and used for further assays.

Human neutrophil isolation

Primary human neutrophils were isolated from venous blood using discarded white blood cell filters provided by the Blood Bank Lab at Boston Children's Hospital. The blood was drawn from healthy donors. The donors from whom the blood was obtained were unidentifiable, and this research did not involve any intervention or interaction with living individuals, nor did it involve identifiable personal information. Therefore, this research is not classified as human subjects research under the HHS human subjects regulations (45 CFR Part 46). Boston Children's Hospital approved the protocol.

Murine peritoneal cavity neutrophil isolation

To recruit neutrophils into the peritoneal cavity, 3% thioglycolate (TG) solution was intraperitoneally injected into wild-type and *Gsdme* KO mice (8-12 weeks old male mice). 6 h after TG injection, peritoneal lavage fluid was collected by three successive washes with 10 ml HBSS containing 0.5% BSA and 15 mM EDTA. Cells were spun down (400 x g, 10 min, RT) and resuspended in 6 ml of 45% Percoll. Collected cells were then layered over discontinuous Percoll/HBSS gradients (62 and 81%) and centrifuged (1500 x g) for 30 min at RT. The interface between the 62% and 81% layers containing neutrophils was harvested and washed with 10 ml of HBSS/EDTA/BSA. Cells were spun down (600 x g, 5 min, RT) and then resuspended in the buffer required for each assay.

Preparation of highly purified neutrophils (HPNs)

To confirm neutrophil-specific GSDME depletion in whole body *Gsdme* KO and neutrophil-specific conditional *Gsdme* KO mice (*Mrp8-cre(+)/Gsdme*$^{\Delta/\Delta}$), we used highly purified neutrophils (HPNs). 8-12 weeks old male mice were used for the isolation. Briefly, mouse neutrophils were first prepared as described above. The isolated neutrophils were further purified using a EasySep™ Mouse Neutrophil Isolation Kit (STEMCELL Technologies, Vancouver, Canada) according to the manufacturer's protocol. Neutrophil purity was determined by flow cytometry. With this approach, we obtain a neutrophil cell suspension with >97% purity.

***In vitro* neutrophil death**

Isolated neutrophils were cultured in RPMI 1640 supplemented with 20% heat-inactivated fetal bovine serum (FBS, Gibco, Thermo Fisher Scientific, Waltham, MA) and penicillin-streptomycin at a density of 2 $\times 10^6$ cells/ml at 37°C in a 5% CO_2 incubator. At each indicated time point, the total cell number was counted using a hemocytometer or FACS with counting beads (CountBright™ Plus Absolute Counting Beads; Thermo Fisher Scientific). The morphology of cultured neutrophils was visualized by light microscopy (400×) at the indicated time points. Dead cells were detected by Annexin V-FITC (2.5 µg/ml) and propidium iodide (PI, 2 µg/ml) staining using an Apoptosis Detection Kit (BD Biosciences) following the manufacturer's protocol. Flow cytometry was performed using a FACSCanto II flow cytometer and analyzed using FlowJo software (BD Biosciences). To investigate neutrophil death in the presence of proinflammatory cytokines, neutrophils were treated with IL-1β, IL-6, and TNF-α (10 ng/ml, Peprotech, Cranbury, NJ) for 20 hours. The cell count and morphology of the dead cells were assessed using the same methods described above.

Western blotting

Western blotting was performed according to our previous publication[4]. Briefly, mouse primary neutrophils were isolated from BM and cultured with or without the indicated inhibitors. Cells were harvested by centrifugation at 400 x g for 5 min. Protein from media supernatants was precipitated with methanol:chloroform:water (4:1:3) followed by centrifugation at 16,000 x g for 5 min, and then the protein and cell pellets were combined and lysed with lysis buffer (2x Laemmli buffer, 2-ME, 1x protease inhibitor cocktail, 0.5 mM DFP, 10 mM PMSF). Samples were incubated in a boiling water bath for 5 mins. 10 µL of lysate was loaded on 5-20% gradient SDS-PAGE gels. Separated proteins were transferred to a Millipore-EPVDF membrane. Membranes were then blocked with 1% milk for an hour followed by incubation with primary GSDME (Abcam, Cambridge, UK; ab215191, EPR19859), GSDMD (Abcam, ab209845, EPR19828), caspase-3 (Cell Signaling Technology, Danvers, MA, #9662), PARP-1 (Cell Signaling Technology, #9542), or β-actin (Cell Signaling Technology, #4970S) antibodies (1:1000 dilution) in 1x TBST overnight at 4°C. After washing in TBST 3 times, membranes were subsequently incubated with goat anti-rabbit HRP-conjugated secondary antibody (Sigma, # A6154,1:5000) for 1 h at room temperature. SuperSignal™ West Femto enhanced chemiluminescent HRP substrate (Thermo Fisher Scientific) was used to detect protein deposited on EPVDF membranes and probed with primary and secondary antibodies. The chemiluminescence signal was captured using Bio-Rad ChemiDoc XP. The relative intensity of each band was quantified using ImageJ.

Peritonitis model

Age- (8-12 weeks) and sex-matched *C57BL/6J* wild-type (WT), *Gsdme* KO, *Mrp8-cre(+)/Gsdme*$^{\Delta/\Delta}$, and *Mrp8-cre(-)/Gsdme*$^{f/f}$ mice were used in the study. Peritonitis was induced by intraperitoneal injection of heat-inactivated *E. coli* (ATCC19138, 10^7 CFU). Mice were humanly euthanized by CO_2 inhalation 48 h after

injection, peripheral blood, BM, and peritoneal lavage fluid were harvested. Peripheral blood was assayed with an automatic hematology analyzer (Mindray). Cells in the BM and peritoneal lavage fluid were collected and resuspended in 200 μL ice-cold PBS, blocked with TruStain FcX™ (anti-mouse CD16/32, BioLegend) at 4℃ for 20 min, and stained with PerCP/Cyanine5.5-CD3 (BioLegend, #100218, Clone 17A2), APC/cy7-B220 (BioLegend, #103224,Clone RA3-6B2), FITC-F4/80 BioLegend, #103224,Clone RA3-6B2), PE-Ly6g (BioLegend, #127608,Clone1A8)), PE/Cy7-Ly6C (BioLegend, #128018,Clone HK1.4), or APC-CD11b (BioLegend, #101212,Clone M1/70) at 4℃ for 20 minutes. Immune cells in the lavage fluid were analyzed using a BD FACSCanto II flow cytometer and FlowJo software (BD Biosciences). Supernatant of peritoneal lavage fluid was collected for cytokine measurements.

Relative death of adoptively transferred neutrophils in inflamed peritoneal cavities

Freshly isolated $CD45.1^+$ and $CD45.2^+$ BM neutrophils from 8-12 weeks old male WT or *Gsdme* KO mice were mixed (1:1, a total of 5×10^6/mouse) and injected i.p. into GFP-expressing *C57BL/6-Tg(CAG-EGFP)10sb/J (B6 ACTb-EGFP)* (Jackson laboratories, Strain #:003291) recipient mice challenged with LPS (5 mg/kg, i.p. injected) for 2.5 h. Peritoneal lavage fluid was harvested 15 h after cell injection. Cells in peritoneal lavage fluid were collected and resuspended in 100 μL ice-cold PBS. Cells were incubated with TruStain FcX™ (anti-mouse CD16/32) Fc blocking antibody at 4℃ for 20 min and stained with PE-CD45.1 (BioLegend, #110708,Clone A20), APC-CD45.2 (BioLegend,#109814,Clone104), or BV421-Ly6g (Biolegend, #127628,Clone.1A8) at 4℃ for 20 min. The relative amounts of $CD45.1^+$ and $CD45.2^+$ neutrophils in GFP-negative cells were analyzed by flow cytometry using a BD FACSCanto II flow cytometer.

CFSE and SNARF-1 labeling of neutrophils for adoptive transfer

Purified neutrophils from the peritoneal cavities of WT or *Gsdme* KO mice (each 8-12 weeks old male) were labeled either with carboxyfluorescein diacetate succinimidyl ester (CFSE, 1 μM) or seminaphthorhodafluor-1 acetate (SNARF-1, 5 μM). The cell density was maintained at 10 million/ml and stained with SNARF-1 or CFSE for 10 min at 37°C. Labelled cells were washed and mixed at a 1:1 ratio (total of 5×10^6 cells/recipient mouse) before being adoptively transferred to the peritoneal cavity of an inflamed host (**Fig.S9D**). After 15 h, the peritoneal cavity was lavaged and the cells were analyzed by FACS to determine the ratio of CFSE/SNARF-1-labeled cells.

Neutrophil death and GSDME cleavage in vivo within inflamed peritoneal cavities

Eight-twelve weeks old male mice were used for the study. Peritonitis was induced by the intraperitoneal injection of heat-inactivated E. coli (ATCC19138, 10^7 CFU). Twenty-four hours after injection, peritoneal lavage fluid was carefully harvested without centrifugation. It was then stained with APC-Ly6g, Annexin V-

FITC, and PI at 4°C for 20 minutes and subsequently fixed with 4% paraformaldehyde at 4°C for 15 minutes. Images were captured using a confocal microscope with a 60x objective. To assess in vivo GSDME cleavage, peritoneal lavage fluid was harvested at 6, 12, 24, and 48 hours after the HIEC injection. Highly purified neutrophils were isolated using the EasySep™ Mouse Neutrophil Isolation Kit. GSDME cleavage was determined by western blotting.

Pneumonia model

Age- (8-12 weeks) and sex-matched *C57BL/6J* wild-type (WT), *Gsdme* KO, *Mrp8-cre(+)/Gsdme*$^{\Delta/\Delta}$, and *Mrp8-cre(-)/Gsdme*$^{f/f}$ mice were used in this study. We induced pneumonia by intra-tracheal injection of the indicated dose of LPS (5-10 mg/kg body weight) or 2.5 µl/g body weight of osmotically balanced 0.1 N HCl as described in our previous publications [5,6]. Briefly, mice were anesthetized using xylazine (10 mg/kg) and ketamine (100-120 mg/kg). The surgical site was prepared by shaving the hair followed by cleaning with iodine- and alcohol-dipped cotton swabs. A small ventral midline incision was made on the neck just above the trachea using sterile scissors. The underlying fatty tissues and muscles were separated to expose the trachea. 50 µl of normal saline containing LPS or HCl was injected directly into lungs via perforation of the trachea using a 24 G insulin syringe (BD Biosciences). Bupivacaine (0.125%) was administered locally to provide analgesia around the surgical site. Mice were humanly euthanized by CO_2 inhalation 24- or 48-hours post LPS or HCl challenge to collect lungs, blood, BM, and the BALF. To establish a pneumonia model induced by *S. aureus*, *Mrp8-cre(-)/Gsdme*$^{f/f}$ and *Mrp8-cre(+)/Gsdme*$^{\Delta/\Delta}$ mice were infected with *S. aureus* (2 x 10^7 CFUs/mouse, ATCC#10390) via the intra-tracheal route. Fresh colonies from overnight-grown bacteria were used for the inoculation. At specified time intervals, mice were euthanized to harvest BALF and lung tissues. To estimate bacterial load, the lungs were homogenized to release bacteria into 1 ml of sterile PBS, then serially diluted and plated onto Tryptic soy agar. The plates were incubated overnight at 37°C. For the survival analysis, each mouse was infected intra-tracheally with 1.5 x 10^8 CFUs of *S. aureus* and monitored over a 7-day period.

Broncho-alveolar fluid collection

BALF was collected as previously described[5]. Briefly, mice were euthanized using CO_2 and then a catheter was inserted into the trachea to flush the lungs. Lungs were lavaged by instilling 0.8 ml of ice-cold PBS/15 mM EDTA. The process was repeated 4 times to collect a total of 2.8-3 ml of lavage fluid. BALF was centrifuged at 400 x g for 5 minutes and supernatant collected for cytokine, LDH, and total protein measurements.

BALF cytokine and total protein measurement

IL-1β (R&D Systems, Minneapolis, MN, SMLB00C), IL-10 (DAKEWE, Shenzhen, China, 1211002), TGF-β (DAKEWE, 1217102), and IL-6 (DAKEWE, 1210602) concentrations in BALF were measured with ELISA

kits following the manufacturers' protocols. Protein concentration was measured using the Bio-Rad protein assay reagent (Bio-Rad Laboratories, Hercules, CA).

LDH measurement

LDH activity was assessed using either the CyQUANTTM LDH Cytotoxicity Assay kit (Thermo Fisher Scientific) or the CytoTox 96® Non-Radioactive Cytotoxicity Assay kit (Promega, Madison, WI) following the manufacturers' protocols. In brief, cell culture supernatants were collected from culture dishes, and BALF was harvested from pneumonia-afflicted mice at specified time points. After centrifuging at 500x g for 5 minutes, the cell-free supernatant was used to measure LDH activity. The sample and reaction mixture were combined in a 1:1 ratio in a 96-well plate and incubated at 37°C for 30 minutes. Following the addition of the stop solution, absorbance was read at 490 nm and 680 nm. LDH activity was calculated by subtracting the absorbance at 680 nm from that at 490 nm. The total LDH from lysates and supernatants served as the Maximum LDH Release Control. LDH release was then determined by comparing the experimental LDH release to the Maximum LDH Release Control.

Lung histopathology

For histopathological assessments, lungs were harvested at the designated time points, fixed in 4% paraformaldehyde (PFA) or 10% formalin (Fisher Scientific, Pittsburg, PA) overnight at RT, and sent to the histopathology core facility at IHCAM or Harvard Medical School. Tissues were gradually dehydrated and embedded in parafilm. The paraffin-embedded lung sections (6 μm) were stained with hematoxylin and eosin (H&E) and analyzed by light microscopy. Non-quantitative histological assessment was performed by a pathologist blinded to the groups. Neutrophil accumulation in alveoli and edema were traced using ImageJ software (NIH) as previously described[5]. Briefly, neutrophil recruitment was calculated as the ratio of pixel area of alveolar space occupied by neutrophils to total pixel area of alveoli. Edematous area was calculated by dividing total pixel area of the whole image by pixel area of all edema-containing regions. While performing histological analysis, the investigators were blinded to the identities of the mice.

Supplemental Movies

Movie S1

Time-lapse imaging of WT neutrophils undergoing programmed death. The experiment was conducted as described in **Fig.1a**. Cells were stained with propidium iodide (PI) (red) and Annexin V (green). Images were acquired every 5 min for 15 h using a 60x oil objective on a Delta Vision Ultra microscope.

Movie S2

Time-lapse imaging of GSDME-deficient neutrophils undergoing programmed death. The experiment was conducted as described in **Fig.1a**. Cells were stained with propidium iodide (PI) (red) and Annexin V (green). Images were acquired every 5 min for 15 h using a 60x oil objective on a Delta Vision Ultra microscope.

Supplemental References

1 Xie, X. *et al.* Single-cell transcriptome profiling reveals neutrophil heterogeneity in homeostasis and infection. *Nat Immunol* **21**, 1119-1133, doi:10.1038/s41590-020-0736-z (2020).

2 Karmakar, M. *et al.* N-GSDMD trafficking to neutrophil organelles facilitates IL-1beta release independently of plasma membrane pores and pyroptosis. *Nat Commun* **11**, 2212, doi:10.1038/s41467-020-16043-9 (2020).

3 Fan, Y. *et al.* Targeting multiple cell death pathways extends the shelf life and preserves the function of human and mouse neutrophils for transfusion. *Sci Transl Med* **13**, doi:10.1126/scitranslmed.abb1069 (2021).

4 Kambara, H. *et al.* Gasdermin D Exerts Anti-inflammatory Effects by Promoting Neutrophil Death. *Cell reports* **22**, 2924-2936, doi:10.1016/j.celrep.2018.02.067 (2018).

5 Hou, Q. *et al.* Inhibition of IP6K1 suppresses neutrophil-mediated pulmonary damage in bacterial pneumonia. *Science Translational Medicine* **10**, doi:10.1126/scitranslmed.aal4045 (2018).

6 Zhu, H. *et al.* Reactive Oxygen Species-Producing Myeloid Cells Act as a Bone Marrow Niche for Sterile Inflammation-Induced Reactive Granulopoiesis. *J Immunol* **198**, 2854-2864, doi:10.4049/jimmunol.1602006 (2017).
