# Supplementary material for: Gasdermin E dictates inflammatory responses by controlling the mode of neutrophil death
Source: Nat Commun. 2024 Jan 9;15:386. doi: 10.1038/s41467-023-44669-y (PMC10776763; doi:10.1038/s41467-023-44669-y)
Supplement: Supplementary file 3 — Description of Additional Supplementary Files [file 41467_2023_44669_MOESM3_ESM.pdf]

Description of Additional Supplementary Files

File Name: Movie S1
Description: Time-lapse imaging of WT neutrophils undergoing programmed death. The experiment was conducted as described in **Fig.1a**. Cells were stained with propidium iodide (PI) (red) and Annexin V (green). Images were acquired every 5 min for 15 h using a 60x oil objective on a Delta Vision Ultra microscope.

File Name: Movie S2
Description: Time-lapse imaging of GSDME-deficient neutrophils undergoing programmed death. The experiment was conducted as described in **Fig.1a**. Cells were stained with propidium iodide (PI) (red) and Annexin V (green). Images were acquired every 5 min for 15 h using a 60x oil objective on a Delta Vision Ultra microscope.
