# Supplementary material for: Gasdermin E dictates inflammatory responses by controlling the mode of neutrophil death
Source: Nat Commun. 2024 Jan 9;15:386. doi: 10.1038/s41467-023-44669-y (PMC10776763; doi:10.1038/s41467-023-44669-y)
Supplement: Supplementary file 6 — Reporting Summary [file 41467_2023_44669_MOESM6_ESM.pdf]

nature portfolio

Corresponding author(s): Hongbo Luo

Last updated by author(s): Dec 5, 2023

Reporting Summary

Nature Portfolio wishes to improve the reproducibility of the work that we publish. This form provides structure for consistency and transparency in reporting. For further information on Nature Portfolio policies, see our Editorial Policies and the Editorial Policy Checklist.

Statistics

For all statistical analyses, confirm that the following items are present in the figure legend, table legend, main text, or Methods section.

n/a	Confirmed	
☐	☒	The exact sample size (*n*) for each experimental group/condition, given as a discrete number and unit of measurement
☐	☒	A statement on whether measurements were taken from distinct samples or whether the same sample was measured repeatedly
☐	☒	The statistical test(s) used AND whether they are one- or two-sided<br>*Only common tests should be described solely by name; describe more complex techniques in the Methods section.*
☐	☒	A description of all covariates tested
☐	☒	A description of any assumptions or corrections, such as tests of normality and adjustment for multiple comparisons
☐	☒	A full description of the statistical parameters including central tendency (e.g. means) or other basic estimates (e.g. regression coefficient) AND variation (e.g. standard deviation) or associated estimates of uncertainty (e.g. confidence intervals)
☐	☒	For null hypothesis testing, the test statistic (e.g. *F*, *t*, *r*) with confidence intervals, effect sizes, degrees of freedom and *P* value noted<br>*Give P values as exact values whenever suitable.*
☒	☐	For Bayesian analysis, information on the choice of priors and Markov chain Monte Carlo settings
☒	☐	For hierarchical and complex designs, identification of the appropriate level for tests and full reporting of outcomes
☒	☐	Estimates of effect sizes (e.g. Cohen's *d*, Pearson's *r*), indicating how they were calculated

Our web collection on statistics for biologists contains articles on many of the points above.

Software and code

Policy information about availability of computer code

Data collection	sc-RNA libraries were constructed using 10X genomics Chromium Single Cell 3'Library & Gel Bead Kit v2. Sequencing data were collected through NOVA-seq6000 from illumina platform.
Data analysis	Cell Ranger v2.2.0 was used to align the sequencing reads (fastq) to the mm10 mouse transcriptome. After gene expression matrix for each sample was created, further analysis was performed using R package Seurat v2.3.4 & v3.0.2,<br>Flow cytometry data were analyzed using Flowjo V10.5.<br>Confocal microscopy data were analyzed using Volocity Software.<br>Statistical data were analyzed using Prism 8 (GraphPad Software, La Jolla, CA).<br>Western blotting data were analyzed using Image J.

For manuscripts utilizing custom algorithms or software that are central to the research but not yet described in published literature, software must be made available to editors and reviewers. We strongly encourage code deposition in a community repository (e.g. GitHub). See the Nature Portfolio guidelines for submitting code & software for further information.

Data

Policy information about availability of data

All manuscripts must include a data availability statement. This statement should provide the following information, where applicable:

- Accession codes, unique identifiers, or web links for publicly available datasets
- A description of any restrictions on data availability
- For clinical datasets or third party data, please ensure that the statement adheres to our policy

All sequencing data have been deposited at NCBI GEO depository and are accessible with the accession number GSE137540 (https://www.ncbi.nlm.nih.gov/geo/query/acc.cgi?acc=GSE137540). The genetically manipulated mice and datasets generated during and/or analyzed during the current study are available from the corresponding authors on reasonable request. Source data are provided with this paper.

Research involving human participants, their data, or biological material

Policy information about studies with human participants or human data. See also policy information about sex, gender (identity/presentation), and sexual orientation and race, ethnicity and racism.

Reporting on sex and gender	In this study, we used human primary neutrophils isolated from venous blood of both males and females.<br>We isolated human primary neutrophils from discarded white blood cell filters (Pall Corporation, East Hills, NY), which were provided by the Blood Bank Lab at the Boston Children's Hospital. We routinely obtain about 1-3 x 108 neutrophils from one filter (450 ml blood from a healthy donor)(59, 91). We have compared the neutrophils that we collected through filter with those obtained by vein puncture and stored in anticoagulant testing tubs, and found that the filtration method does not impair neutrophil function (e.g. chemotaxis, phagocytosis, H2O2 production, and the time course of cell death) (59).<br>We always coordinate with the blood bank technicians to make sure that the filters are prepared using the same standard procedure and delivered to us in time. All blood was drawn from healthy blood donors. Neither I nor my collaborators could identify the subjects from whom the blood was obtained (the gender information was provided for research purpose) and this research did not involve intervention or interaction with living individuals, or identifiable private information. Thus this research is not human subjects research under the HHS human subjects regulations (45 CFR Part 46). This protocol is approved by the Children's Hospital Institutional Review Board (IRB) (Protocol #IRBP00020263).
Reporting on race, ethnicity, or other socially relevant groupings	N/A
Population characteristics	N/A
Recruitment	N/A
Ethics oversight	N/A

Note that full information on the approval of the study protocol must also be provided in the manuscript.

Field-specific reporting

Please select the one below that is the best fit for your research. If you are not sure, read the appropriate sections before making your selection.

☒ Life sciences ☐ Behavioural & social sciences ☐ Ecological, evolutionary & environmental sciences

For a reference copy of the document with all sections, see nature.com/documents/nr-reporting-summary-flat.pdf

Life sciences study design

All studies must disclose on these points even when the disclosure is negative.

Sample size	We chose sufficient sample size to establish that the values compared were derived from normal distributions. Sample sizes are always indicated in figure legends or methods section.
Data exclusions	No data was excluded from the analysis.
Replication	Experiments were performed at least three times independently and successfully reproduced. Reproducibility of the experiments and significances of the results are shown in details in figure legends or method.
Randomization	Mice were age matched, and randomized and blindly coded for group assignment and data collection.
Blinding	The investigators were blinded to group allocation during data collection and analysis.

Reporting for specific materials, systems and methods

We require information from authors about some types of materials, experimental systems and methods used in many studies. Here, indicate whether each material, system or method listed is relevant to your study. If you are not sure if a list item applies to your research, read the appropriate section before selecting a response.

Materials & experimental systems

n/a	Involved in the study
☐	☒ Antibodies
☐	☒ Eukaryotic cell lines
☒	☐ Palaeontology and archaeology
☐	☒ Animals and other organisms
☒	☐ Clinical data
☒	☐ Dual use research of concern
☒	☐ Plants

Methods

n/a	Involved in the study
☒	☐ ChIP-seq
☐	☒ Flow cytometry
☒	☐ MRI-based neuroimaging

Antibodies

Antibodies used

Flow cytometry antibodies: APC-Ly6g (BioLegend, #108423, Clone 1A8), Percp-Cy5.5-F4/80 (BioLegend, # 157318, Clone BM8), PE-Cy7-CD11b (BioLegend, #101216, Clone M1/70), APC-Cy7-Ly6c (BioLegend , #128026, Clone HK1.4), PE-CD45.1(BioLegend, #110708,Clone A20), APC-CD45.2 (BioLegend,#109814,Clone104),BV421-Ly6g (Biolegend, #127628,Clone.1A8), TruStain FcX™ (anti-mouse CD16/32, BioLegend), PerCP/Cyanine5.5-CD3 (BioLegend, #100218, Clone 17A2), APC/cy7-B220 (BioLegend, #103224,Clone RA3-6B2), FITC-F4/80 BioLegend, #103224,Clone RA3-6B2), PE-Ly6g (BioLegend, #127608,Clone1A8)), PE/Cy7-Ly6C (BioLegend, #128018,Clone HK1.4), APC-CD11b (BioLegend, #101212,Clone M1/70)

Western blot antibodies (Primary): GSDME (Abcam, Cambridge, UK; ab215191), GSDMD (Abcam, ab209845), caspase-3 (Cell Signaling Technology, Danvers, MA, 9662), PARP-1 (Cell Signaling Technology, 9542), and β-actin (Cell Signaling Technology, 4970S) antibodies.
Western blot antibodies (Secondary): HRP-conjugated goat anti-rabbit antibody (Sigma-Aldrich; Cat: A6154),

Validation

All antibodies used for flow cytometry and western blot are well-established antibodies. Staining patterns were consistent with the manufacturer product information as well as published data.

Eukaryotic cell lines

Policy information about cell lines and Sex and Gender in Research

Cell line source(s)

HEK293T is purchased from ATCC (CRL-3216). It is an epithelial-like cell that was isolated from the kidney of a patient.

Authentication

The cell lines were authenticated based on morphology comparison with ATCC culture collection database images

Mycoplasma contamination

No infection

Commonly misidentified lines
(See ICLAC register)

No commonly misidentified cell lines were used

Animals and other research organisms

Policy information about studies involving animals; ARRIVE guidelines recommended for reporting animal research, and Sex and Gender in Research

Laboratory animals

C57BL/6J wild-type (WT, Strain #:000664), B6.Cg-Tg(Mrp8-cre,-EGFP)1Ilw/J (Strain #:021614), and C57BL/6-Tg(CAG-EGFP)10sb/J (B6 ACTb-EGFP) (Strain #:003291)mice were purchased from Jackson Laboratories (Bar Harbor, ME). Mice were kept in a specific pathogen-free cage system under a 12/12 h light/dark cycle with free access to food and water.

Gsdme KO mice were generated on a pure C57BL/6J background using the CRISPR/Cas9 system targeting exon 3. Gsdmeflox/flox mice were produced by inserting loxP into both ends of exon 3. Neutrophil-specific conditional Gsdme KO mice (Mrp8-cre(+)/GsdmeΔ/Δ) were generated by crossing Gsdmeflox/flox mice with B6.Cg-Tg(Mrp8-cre,-EGFP)1Ilw/J mice. Heterozygous F1 parents were used for mating to generate Mrp8-cre(+)/GsdmeΔ/Δ and Mrp8-cre(-)/Gsdmef/f littermates.
All 8-12 weeks mice are used for experiments

Wild animals

No wild animal was used in this study.

Reporting on sex

female and male

Field-collected samples	No field collected samples were used in this study
Ethics oversight	All animals were housed in specific pathogen-free barrier facilities and used in accordance with protocols approved by the Boston Children’s Hospital Animal Care and Use Committee and the Institutional Animal Care and User Committee at the Institute of Hematology, Chinese Academy of Medical Sciences.

Note that full information on the approval of the study protocol must also be provided in the manuscript.

Flow Cytometry

Plots

Confirm that:

☒ The axis labels state the marker and fluorochrome used (e.g. CD4-FITC).

☒ The axis scales are clearly visible. Include numbers along axes only for bottom left plot of group (a 'group' is an analysis of identical markers).

☒ All plots are contour plots with outliers or pseudocolor plots.

☒ A numerical value for number of cells or percentage (with statistics) is provided.

Methodology

Sample preparation	Bone marrow and peritoneal cavity neutrophils were isolated and cultured for different time points. Cells were harvested, stained with Annexin V-FITC and PI, and analyzed via flow cytometry.<br>BM and BALF cells were collected and then centrifuged at 400 x g for 5 min. Cells were suspended in 200 µL ice-cold PBS, blocked with TruStain FcX™ (anti-mouse CD16/32, BioLegend, San Diego, CA) at 4°C for 20 min, and stained with PE/Cyanine7-CD45, APC/Cyanine7-F4/80, APC-CD11b, PE-Ly6G, and FITC-Ly6C antibodies (BioLegend) at 4°C for 20 min.
Instrument	FACS CantoII (BD Biosciences, Franklin Lakes, NJ)
Software	BD FACSDIVA
Cell population abundance	Abundance of the cell population in the detected samples were indicated in Fig.2,4 and 6 and Extended Data Fig. 3
Gating strategy	Intact cells were gated according to the FSC-A and SSC-A. Doublets were excluded by FSC-H and FSC-A. Gating strategies for specific cell populations were illustrated in Fig.2,4 and 6 and Extended Data Fig. 3

☒ Tick this box to confirm that a figure exemplifying the gating strategy is provided in the Supplementary Information.
